# Offensive role of the *Bacillus* extracellular matrix in driving metabolite-mediated dialog and adaptive strategies with the fungus *Botrytis*

**DOI:** 10.1093/ismejo/wraf277

**Published:** 2025-12-18

**Authors:** Alicia I Pérez-Lorente, Carlos Molina-Santiago, David Vela-Corcía, Paolo Stincone, Jesús Hierrezuelo, Montserrat Grifé, Abzer K Pakkir Shah, Antonio de Vicente, Daniel Petras, Diego Romero

**Affiliations:** Instituto de Hortofruticultura Subtropical y Mediterránea “La Mayora”, Universidad de Málaga-Consejo Superior de Investigaciones Científicas, Bulevar Louis Pasteur, 49, Málaga, 29010, Spain; Departamento de Microbiología, Universidad de Málaga, Bulevar Louis Pasteur 31 (Campus Universitario de Teatinos), Málaga, 29071, Spain; Instituto de Hortofruticultura Subtropical y Mediterránea “La Mayora”, Universidad de Málaga-Consejo Superior de Investigaciones Científicas, Bulevar Louis Pasteur, 49, Málaga, 29010, Spain; Departamento de Microbiología, Universidad de Málaga, Bulevar Louis Pasteur 31 (Campus Universitario de Teatinos), Málaga, 29071, Spain; Instituto de Hortofruticultura Subtropical y Mediterránea “La Mayora”, Universidad de Málaga-Consejo Superior de Investigaciones Científicas, Bulevar Louis Pasteur, 49, Málaga, 29010, Spain; Departamento de Microbiología, Universidad de Málaga, Bulevar Louis Pasteur 31 (Campus Universitario de Teatinos), Málaga, 29071, Spain; University of Tuebingen, Cluster of Excellence, Interfaculty Institute of Microbiology and Infection Medicine, Auf der Morgenstelle 28, Tuebingen, 72074, Germany; Instituto de Hortofruticultura Subtropical y Mediterránea “La Mayora”, Universidad de Málaga-Consejo Superior de Investigaciones Científicas, Bulevar Louis Pasteur, 49, Málaga, 29010, Spain; Departamento de Microbiología, Universidad de Málaga, Bulevar Louis Pasteur 31 (Campus Universitario de Teatinos), Málaga, 29071, Spain; Instituto de Hortofruticultura Subtropical y Mediterránea “La Mayora”, Universidad de Málaga-Consejo Superior de Investigaciones Científicas, Bulevar Louis Pasteur, 49, Málaga, 29010, Spain; Departamento de Microbiología, Universidad de Málaga, Bulevar Louis Pasteur 31 (Campus Universitario de Teatinos), Málaga, 29071, Spain; University of Tuebingen, Cluster of Excellence, Interfaculty Institute of Microbiology and Infection Medicine, Auf der Morgenstelle 28, Tuebingen, 72074, Germany; Departamento de Microbiología, Universidad de Málaga, Bulevar Louis Pasteur 31 (Campus Universitario de Teatinos), Málaga, 29071, Spain; University of Tuebingen, Cluster of Excellence, Interfaculty Institute of Microbiology and Infection Medicine, Auf der Morgenstelle 28, Tuebingen, 72074, Germany; University of California Riverside, Department of Biochemistry, 169 Aberdeen Drive, Riverside, CA 92507, United States; Instituto de Hortofruticultura Subtropical y Mediterránea “La Mayora”, Universidad de Málaga-Consejo Superior de Investigaciones Científicas, Bulevar Louis Pasteur, 49, Málaga, 29010, Spain; Departamento de Microbiología, Universidad de Málaga, Bulevar Louis Pasteur 31 (Campus Universitario de Teatinos), Málaga, 29071, Spain

**Keywords:** Bacillus, biofilm, metabolomics, bacterial–fungal interactions, microbial ecology, secondary metabolism

## Abstract

Bacterial–fungal interactions have traditionally been attributed to secondary metabolites, but the role of the bacterial extracellular matrix in shaping these relationships has remained unclear. Here, we demonstrate that the extracellular matrix protein TasA is a key mediator in the antagonistic interaction between Bacillus subtilis and *Botrytis cinerea*. TasA enables *Bacillus* to tightly adhere to fungal hyphae, disrupts the β-glucan layer, and compromises fungal cytoskeletal integrity synergistically with fengycin, which causes cytological damage. Additionally, TasA acts as a carrier for bacillaene, amplifying its fungistatic activity. In response, *Botrytis* mounts a multifaceted defense, enzymatically degrading fengycin, producing antibacterial oxylipins, and activating adaptive programs such as hyphal branching and chlamydospore formation. Our findings reveal the previously unrecognized role of extracellular matrix components in fungal suppression and the modulation of fungal adaptive responses. This study reveals the complex interplay between microbial aggression and defense, providing new insights into the ecological dynamics of microbial competition and coexistence.

## Introduction

Microbial interactions shape microbial community dynamics and impact ecosystems, particularly within the plant microbiome [1–3]. These interactions range from cooperative to competitive and are often mediated by secondary metabolites, signaling molecules, and biofilms that influence plant health [4]. Beneficial microbes support nutrient acquisition, plant defense, and stress tolerance but compete for limited resources [5, 6] with detrimental pathogens, including fungal species such as *Botrytis cinerea* [7–9].

Bacillus subtilis is a gram-positive bacterium known for its ability to promote plant growth and effectively antagonize fungal pathogens such as *B. cinerea*, the causative agent of gray mold disease, which significantly impacts crops and leads to economic losses [7–9]. The antagonistic activity of *B. subtilis* stems from its production of antifungal lipopeptides, such as fengycin and surfactin, and apparently robust biofilm formation capacity [10]. The extracellular matrix (ECM) of *B. subtilis* comprises mainly but not exclusively exopolysaccharides (EPSs), TasA, TapA, and BslA [11, 12], which provide structural integrity and participate in interactions with plants [13]; bacteria such as *Pseudomonas* species [14]; and fungal species such as *Aspergillus niger* [15, 16] or *Rhizophagus irregulari* [17]. However, the exact role of the ECM in interactions with phytopathogenic fungi remains poorly understood.

Traditionally, the *B. subtilis–B. cinerea* interaction has been viewed as being statically antagonistic. However, microbial interactions are dynamic, with both organisms undergoing metabolic and transcriptomic changes [18]. While *B. subtilis* suppresses *B. cinerea* growth, a portion of the fungal population adapts and survives [19, 20]. This study demonstrates the multifaceted involvement of the ECM, beyond adhesion, in this interkingdom chemical crosstalk and in antagonistic interactions, with TasA and fengycin as key components driving fungal modulation and ecological balance within microbial ecosystems.

## Materials and methods

### Strains, media, and culture conditions

A complete list of the bacterial strains used in this study is shown in Table S1. *Bacillus* cultures were grown in liquid lysogeny broth (LB; 1% tryptone, 0.5% yeast extract and 0.5% NaCl) at 30°C, while *Escherichia coli* were cultivated at 37°C, both with shaking on an orbital platform. The medium was adjusted to pH 7 prior to sterilization. Antibiotics were added at standard concentrations whenever required. Isolate B05.10 of the necrotrophic fungus *B. cinerea* was cultured on potato dextrose agar (PDA, Oxoid) at 20°C under illumination with fluorescent light at a photofluency rate of 12 μmol m^−2^ s^−1^ and a 12/12 h photoperiod. Conidia were collected from these cultures using sterile distilled water and filtered through a 40-μm cell strainer to eliminate hyphal fragments. *Escherichia coli* DH5α was used for cloning and plasmid replication. *E. coli* BL21 (DE3) and BL21(AI) were used for protein purification.

*Bacillus subtilis* mutants

*B. subtilis* mutants were obtained through SPP1-mediated phage transduction following standard protocol [21]. To generate double mutant strains, phage lysates were first prepared from the ∆tasA and ∆eps single mutant strains and then used to transduce a ∆pps background mutant. All the mutants were confirmed through polymerase chain reaction amplification and antibiotic resistance assays.

### Construction of fluorescence-labeled strains

The fluorescence labeling plasmid pKM008V was constructed for Bacillus subtilis strains. In brief, a 300 bp fragment of the Pveg promoter was extracted from pBS1C3 using restriction enzymes EcoRI and HindIII. The Pveg promoter was selected because of its role as a constitutive promoter in *B. subtilis.* After purification, the fragment was inserted into the pKM003 (YFP) or pDR183 (CFP) plasmid, which had been digested with the same enzymes. The plasmid was introduced into *B. subtilis* 168 by natural competence. Transformants were selected by plating on LB agar supplemented with antibiotics. To fluorescently label the extracellular matrix mutants, YFP or CFP was transferred from *B. subtilis* 168 using SPP1 phage transduction as previously described [21].

### Bacterial–fungal competition assay

*B. cinerea* was precultured by growing a germinated conidial suspension in potato dextrose broth (PDB, Oxoid) at 28°C for 24 h at 150 rpm. *B. subtilis* strains were grown on LB plates overnight at 28°C and the resulting colonies were grown overnight in 5 ml of LB at 28°C on an orbital platform. Then, 10–15 germinated hyphae (microcolonies) were mixed with 200 μl of bacterial culture grown in 6-well plates with PDB at 28°C overnight at 150 rpm.

### Evaluation of *Bacillus* adhesion

To analyze the adhesion of different *Bacillus* ECM mutants to *Botrytis* hyphae, *Botrytis* was mixed with precultures of the different *Bacillus* strains constitutively labeled with YFP or CFP. After overnight incubation at 28°C in PDB, the *Botrytis* clumps were washed three times with water to remove unattached *Bacillus* cells. The washed hyphae were subsequently placed onto agarose-coated slides. For the *Bacillus* strains labeled with CFP, excitation and emission detection were performed at the corresponding wavelengths (excitation at 405 nm and emission detection between 450 and 550 nm), and likewise for YFP-labeled strains (excitation at 512 nm and emission detection between 520 and 600 nm). Images were obtained using a Leica Stellaris 8 confocal microscope with a 63× NA 1.3 Plan APO oil-immersion objective. For each experiment, the laser settings, scan speed, PMT or HyD detector gain, and pinhole aperture were kept constant across all acquired images. To count CFUs adhered to the fungus with each ECM mutant, the fungal mass was separated from the liquid bacterial culture by filtering with a 40 μm nylon filter to retain the fungus. The fungal mass was then washed once with Milli–Q (MQ) water to remove the free bacterial cells that were not attached to the fungi. The washed fungal mass was collected in a microtube and weighed to normalize the results to the mass of fungus. A pestle was then used to break the fungal structures and release the bacteria adhering to the fungus. The content was resuspended, and serial dilutions were performed. Finally, CFUs were counted and normalized to the fungal mass.

### Transmission electron microscopy

*B. cinerea* samples were fixed in 2.5% (v/v) glutaraldehyde and 4% (v/v) paraformaldehyde overnight at 4°C. After three rinses with fixation buffer, samples were post-fixed in 1% osmium tetroxide for 90 min at room temperature. They were then washed twice and 15 min of stepwise dehydration in an ethanol series (30%, 50%, 70%, 90%, and 100% twice). Between the 50% and 70% ethanol steps, samples were incubated overnight at 4°C in 2% uranyl acetate prepared in 50% ethanol. Following dehydration, the samples were gradually embedded in low-viscosity Spurr’s resin as follows: resin:ethanol, 1:1, 4 h; resin:ethanol, 3:1, 4 h; and pure resin, overnight. The sample blocks were embedded in capsule molds containing pure resin for 72 h at 70°C. The samples were left to dry and visualized under a FEI TALOS F200X.

### Confocal laser scanning microscopy

Bacterial cell death within the colonies was assessed utilizing the LIVE/DEAD BacLight Bacterial Viability Kit (Invitrogen). Equal volumes of the kit components were mixed and 2 μl of this solution was applied to 1 ml of bacterial suspension for staining. To visualize live or dead bacteria in the samples, sequential acquisitions were performed. Live bacteria were imaged using excitation at 488 nm, and emission was recorded between 499 and 554 nm, whereas dead bacteria were imaged through a subsequent acquisition via excitation at 561 nm and emission recorded between 592 and 688 nm.

Intracellular ROS levels were detected by staining with dihydrorhodamine 123 (DHR123; Sigma). After adding DHR123 to a final concentration of 2 μg/ml, samples were incubated for 5 min at room temperature. The cells were counterstained with the lipophilic dye FM4–64 (Thermo Fisher) to stain the plasma membrane. Images were obtained using a Leica Stellaris 8 confocal microscope with a 63x NA 1.3 Plan APO oil-immersion objective, with excitation at 488 nm and emission detection between 510 and 580 nm (for DHR123 fluorescence emission) and between 670 and 850 nm (for FM4–64 fluorescence emission). Image processing was performed using FIJI/ImageJ software [22]. Image processing was conducted keeping laser settings, scan speed, detector gain and pinhole size constant across experiments.

### Protein purification

Recombinant His6-tagged TasA was purified as previously reported [23]. In summary, E. coli BL21 (DE3) cells carrying the pET22b-tasA plasmid were cultivated overnight in 5 ml LB medium at 37°C with agitation (150 rpm). Cultures were reinoculated 1:100 into fresh LB and grown until reaching an OD_600_ of 0.7–0.8, when protein expression was induced with 1 mM IPTG. Induced cultures were incubated overnight at 28°C with shaking to promote inclusion body formation. The next day, the cells were harvested via centrifugation (7000 × g, 30 min, 4°C) and stored at −80°C until purification. After thawing, the pellet was resuspended in buffer A (Tris 50 mM, 150 mM NaCl; pH 8) supplemented with 0.2 mg/ml lysozyme, 1 mM PMSF, and 10x Cell Lysis Reagent (Sigma) and incubated for 1 h at 37°C. Solubilization was achieved by adding 6 M guanidine hydrochloride (GuHCl) to saturation and incubating at 60°C overnight. The lysate was sonicated on ice (3 × 60 s, 60% amplitude) and centrifuged (110 000 × g, 1 h, 16°C). The resulting supernatant was passed through a 0.45-μm filter prior to affinity chromatography. The protein was purified using an AKTA Start FPLC system (GE Healthcare). The suspension was loaded into a HisTrap HP 5 ml column (GE Healthcare) previously equilibrated with binding buffer (50 mM Tris, 0.5 M NaCl, 20 mM imidazole, and 8 M urea; pH 8). Next, the protein was eluted from the column with elution buffer (50 mM Tris, 0.5 M NaCl, 500 mM imidazole, and 8 M urea; pH 8). Subsequently, fractions were passed through a HiPrep 26/10 desalting column (GE Healthcare) and exchanged into either 20 mM Tris or 50 mM NaCl buffer, depending on the downstream assays.

For chitosanase, recombinant His_6_-tagged Csn was purified using a modified protocol for heterologous expression. E. coli harboring the expression plasmid were grown in 250 ml LB supplemented with ampicillin to an OD_600_ of 0.7, then induced with IPTG and incubated under optimal conditions prior to harvesting (10 000 × g, 5 min). The cell pellets were lysed in washing buffer containing 50 mM Na_3_PO_4_ (pH 8), 500 mM NaCl, 10 mM imidazole, 1 mM PMSF, 0.2 mg/ml lysozyme, and 10x CelLytic (Sigma–Aldrich) and incubated with shaking at room temperature for 30 min. Cells were disrupted by sonication on ice (3 × 60 s) and clarified by centrifugation and filtration.

The lysate was loaded onto a HisTrap HP column (GE Healthcare) on an AKTA Start FPLC system equilibrated with washing buffer. The protein was eluted via elution buffer (20 mM Na*3*PO4, 500 mM NaCl, and 500 mM imidazole; pH 8). The purified Csn protein was dialyzed with a HiPrep 26/10 desalting column (GE Healthcare) against 50 mM Tris–HCl and 50 mM NaCl (pH 7.4), and stored at −80°C until use.

### EPS extraction and purification

For EPS production, the *B. subtilis* ΔtasA strain was employed. An overnight preculture in Ty medium was prepared and 1 ml of this culture was inoculated into 200 ml of fresh Ty medium for static incubation in 24-well plates at 30°C for 5 days. Cells were harvested by centrifugation (8000 × g, 10 min) and washed with distilled water. The resulting pellets were resuspended in phosphate buffered saline (PBS) and gently sonicated to detach EPS from the cells. Soluble fractions were stirred on ice and treated with trichloroacetic acid (TCA, final concentration 100%) to precipitate contaminating proteins. After overnight incubation at 4°C, proteins were removed by centrifugation (10 000 × g, 20 min). EPS was then precipitated from the clarified supernatant by adding five volumes of cold ethanol under constant stirring and incubating overnight at 4°C. The precipitated material was recovered by centrifugation (10 000 × g, 20 min), dialyzed against distilled water overnight (Spectra/Por membrane, MWCO 3.5 kDa), filtered, and subjected to size-exclusion chromatography (HiPrep 16/60 Sephacryl S-300HR) using Milli-Q water as the eluent. Fractions were analyzed with the phenol–sulfuric acid assay, and absorbance at 490 nm was recorded in a FLUOstar Omega plate reader (BMG LabTech). Carbohydrate-containing fractions were combined and lyophilized for downstream analyses.

### Immunolocalization of TasA by Confocal Laser Scanning Microscopy (CLSM)

To evaluate the interaction of purified tagged TasA with *Botrytis* hyphae, 3 μm of the protein was added and the mixture incubated overnight. Next, the samples were applied to well slides treated with 0.1% poly-L-lysine (Sigma–Aldrich) and incubated for 2 h. After the samples were removed, they were fixed with fixation buffer (3% paraformaldehyde and 0.1% glutaraldehyde diluted in PBS) for 10 min. The wells were subsequently rinsed twice with PBS and incubated for 1 h in blocking buffer [3% w/v bovine serum albumin (BSA) and 0.2% v/v Triton X-100 in PBS]. Following removal of the buffer, the wells were treated with the primary antibody (anti-His) at a concentration of 1:100 diluted in blocking buffer and incubated for 3 h. The wells were then washed three times with washing buffer (0.2% w/v BSA and 0.05% v/v Triton X-100 in PBS), with each incubation lasting for 5 min. The wells were subsequently incubated for 2 h with the secondary antibody goat anti-rabbit IgG-Atto488 at a dilution of 1:200 in blocking buffer. The samples were washed once with washing buffer and twice with PBS, with each incubation lasting for 5 min. The immunostained samples were fixed for 5 min with fixation buffer, followed by rinsing with PBS three times. As a negative control, immunostaining was performed without incubation with the primary antibody. Visualization of immunostaining was carried out using confocal laser scanning microscopy. Fluorescence of Atto-488 was recorded using excitation at 488 nm and emission between 497–572 nm. Alexa Fluor 647 was detected with excitation at 561 nm and emission in the range of 576–686 nm.

### TasA immunolabeling assay by transmission electron microscopy

To identify the specific localization of TasA on the cell wall of *Botrytis*, we first added 3 μm purified TasA to a *Botrytis* culture and incubated it overnight. next, the cells were fixed and embedded as described in the transmission electron microscopy (TEM) section. For immunolabeling assays, carbon-coated copper grids were placed onto the samples of *Botrytis*. After 2 h of incubation, the grids were washed in PBS for 5 min and subsequently blocked with Pierce protein-free (TBS) blocking buffer (Thermo Fisher) for 30 min. An anti-TasA primary antibody was used at a 1:150 dilution in blocking buffer, and the grids were deposited over the drops of antibody solution and incubated for 1 h at room temperature. The samples were washed three times with TBS-T (50 mM Tris–HCl, 150 mM NaCl, pH 7.5, and 0.1% Tween 20) for 5 min and then exposed to a 10-nm-diameter immunogold-conjugated secondary antibody (10 nm goat anti-rabbit conjugate, BBI solutions) for 1 h at a 1:50 dilution. The samples were then washed twice with TBS-T and once with water for 5 min each time. Finally, the preparations were treated with 2% glutaraldehyde for 10 min, washed in water for 5 min, counterstained with 1% uranyl acetate for 15 s, and rinsed once with water for 15 s. The grids were allowed to dry, and imaging was performed using a JEOL JEM-1400 transmission electron microscope operated at 80 kV.

### Magnetic resonance imaging assays

*B. cinerea* cells (either treated with purified TasA or protein buffer as control) were incubated for 24 h in PDB. Following incubation, all samples were embedded in 1.5% agar in Falcon tubes to ensure stable positioning for imaging. Magnetic resonance imaging (MRI) experiments were conducted using a 9.4 T Bruker Biospec system equipped with 400 mT/m gradients and an Avance III console (Bruker BioSpin, Ettlingen, Germany). High-resolution T2-weighted images were acquired using a turbo-RARE sequence with the following parameters: TE = 33 ms, TR = 500 ms, 2 averages, a field of view (FOV) of 3.2 cm, a matrix size of 384 × 384, an in-plane resolution of 78 μm, and a slice thickness of 1 mm. These settings allowed detailed observation of structural and textural alterations in *Botrytis* samples following TasA exposure.

### Plant infection assays

Assays of *B. cinerea* infection were carried out in 5–6-week-old plants. Fungal conidia were collected from cultures grown under illumination in sterile distilled water and filtered through a 40 μm cell strainer to remove the remaining hyphae. For inoculation, the conidial suspension was adjusted to 10^5^ conidia/ml in grape juice (100% pure organic). Leaves were inoculated by applying 5 μl droplets of conidial suspension. The pots were covered with a plastic dome and placed in a growth chamber. After 24 h, the plastic dome was briefly removed to administer either TasA treatment or buffer in the case of control plants. Leaf images were acquired 72 h post-inoculation, and lesion size was quantified using ImageJ software.

### Ribonucleic acid isolation and sequencing

Ribonucleic acid (RNA) from *B. cinerea* was extracted from clumps disrupted with liquid nitrogen. After disruption, the suspensions of the pellets were resuspended in TRIzol reagent (Invitrogen). Total RNA extraction was then performed as indicated by the manufacturer. DNA removal was carried out by treatment with Nucleo-Spin RNA Plant (Macherey–Nagel). The integrity and quality of the total RNA were assessed with an Agilent 2100 Bioanalyzer (Agilent Technologies) via electrophoresis. The removal of rRNA was performed using the RiboZero rRNA Removal (Bacteria) Kit from Illumina, and 100-bp single-end read libraries were prepared via the TruSeq Stranded Total RNA Kit (Illumina). The libraries were sequenced using a NextSeq 550 System (Illumina). The raw reads were preprocessed with SeqTrimNext The raw reads were preprocessed with SeqTrimNext [24] using specific high-throughput sequencing parameters. This preprocessing removed low-quality, ambiguous and low-complexity stretches; linkers; adapters; vector fragments; and contaminated sequences while keeping the longest informative parts of the reads. Reads shorter than 25 bp were discarded. Clean reads were mapped to the *B. subtilis* reference genome using Bowtie [25]. Alignment files were processed in BAM format and subsequently sorted and indexed with SAMtools v1.484 [26]. Gene-level read counts were obtained using Sam2counts (https://github.com/vsbuffalo/sam2counts). Differential gene expression was assessed with DEgenes Hunter, which combines p-values from edgeR [27] and DEseq2 via Fisher’s method. Resulting p-values were corrected using the Benjamini–Hochberg false discovery rate (FDR) method, and genes with an adjusted *P* < .05 and log2 fold-change >1 or < −1 were considered significantly differentially expressed.

### Metabolite extraction from liquid culture

The cultures were centrifuged to separate cell pellets from the supernatant. For metabolite extraction from the cellular fraction, 1 ml of 80% methanol was added, and a tissue-lyser was used for 10 min to disrupt the cells. The mixture was then centrifuged at maximum speed, and the supernatant was transferred to a new tube. The methanol was evaporated using a speed vacuum concentrator and stored at −20°C until analysis by liquid chromatography-mass spectrometry (LC–MS). For the supernatant-derived metabolites, ethyl acetate was added at a 1:1 ratio, by volume, with the supernatant. The mixture was vortexed and rotated for 30 min. Subsequently, 4 ml of the upper phase was transferred to a new tube. The supernatant was evaporated using a speed vacuum concentrator and stored at −20°C until further analysis via LC–MS.

### Liquid chromatography–tandem mass spectrometry

Nontarget metabolomic profiling Untargeted metabolomic profiling was carried out using ultra-high-performance liquid chromatography (UHPLC) coupled to a Q Exactive HF mass spectrometer, as previously described [28]. Briefly, UHPLC separation was performed using a C18 core–shell column (Kinetex, 50 × 1 mm, 1.7 μm particle size, 100 A pore size; Phenomenex, Torrance, USA). The mobile phases used were solvent (A), containing H2O (LC/MS grade, Fisher Scientific) + 0.1% formic acid (FA), and solvent (B), containing acetonitrile (LC/MS grade, Fisher Scientific) + 0.1% FA. Following sample injection, a 5 min linear gradient was applied for small-molecule elution, with a flow rate of 150 μl/min (microflow mode). The following separation conditions were used: 0–4 min from 5% to 50% solvent (B), 4–5 min from 50 to 99% B, followed by a 2 min wash step at 99% B and a 3 min re-equilibration phase at 5% B. Mass spectrometry was performed in positive ion mode using HESI. The parameters were set as: sheath gas flow 30 L/min, auxiliary gas 10 L/min, sweep gas 2 L/min, spray voltage 3.5 kV, capillary temperature 250°C, S-lens RF level 50 V, and auxiliary gas heater temperature 200°C. Full MS survey scans were acquired across an m/z range of 120–1800 with a resolution of 45 000, automatic gain control (AGC) of 1 × 10^6^, maximum injection time of 100 ms, and one microscan. data-dependent acquisition (DDA) MS/MS spectra acquisition was performed in DDA mode with TopN set to 5; as a consequence, the five most abundant precursor ions of the survey MS scan were subjected to MS/MS fragmentation. The resolution of the MS/MS spectra was set to 15 000, the AGC target was 5E5, and the maximum injection time was 50 ms. The quadrupole precursor selection width was set to 1 m/z. Normalized collision energy was applied in stepped mode at 25, 35, and 45°C. MS/MS scans were triggered in apex mode within 2–15 s from their first occurrence in a survey scan. Dynamic precursor exclusion was set to 5 s.

### Feature-based molecular networking and spectral library search

Following LC–MS/MS acquisition, raw spectra were converted to *.mzML* files using MSConvert (ProteoWizard). MS1 and MS/MS feature extraction was performed with Mzmine3 [29]. For MS1 spectra, an intensity threshold of 1E5 was used, and for MS/MS spectra, an intensity threshold of 1E3 was used. For MS1 chromatogram building, a 10-ppm mass accuracy and a minimum peak intensity of 5E5 were set. Extracted ion chromatograms were deconvoluted with the baseline cutoff algorithm (threshold 1E5), and matched to MS/MS spectra within 0.02 m/z and 0.2 min retention time windows. Isotope peaks were grouped, and features from different samples were aligned with 10 ppm mass tolerance and 0.1-min retention time tolerance. MS1 features without MS2 features assigned were filtered out of the resulting matrix, as were features that did not contain isotope peaks and that did not occur in at least three samples. After filtering, the gaps in the feature matrix were filled with a relaxed retention time tolerance of 0.2 min and a 10 ppm mass tolerance. Finally, the feature table was exported as a .csv file, and the corresponding MS/MS spectra were exported as .mgf files. Contaminant signals detected in blank samples were filtered out, retaining only those with a blank-to-sample abundance ratio below 30%. For feature-based molecular networking and spectrum library matching, the .mgf file was uploaded to GNPS [30, 31].

For network construction, parameters were set to a cosine score ≥ 0.7, precursor and fragment ion tolerances of 0.01 Da, a minimum of six matched fragment peaks, and a cluster size threshold of one (MS cluster-off). Library searches required at least five matched fragment peaks, with analog searches allowing a maximum precursor mass difference of 100 m/z at a cosine score of ≥0.7. Networks were visualized in Cytoscape v3.9.1102 [32].

To enhance the chemical structural information in the molecular network, the generated .mgf file from MzMine3 was placed into Sirius 4 for chemical class and structure prediction [33]. Data were compared and classified against databases present in SIRIUS (Bio Database, GNPS, Natural Products, PubChem, PubMed). For molecular formula identification, the MS2 mass accuracy was set to 3 ppm. Chemical class annotations were performed with CSI: FingerID [34] and CANOPUS [35]. Mirror plots were generated using GNPS and https://metabolomics-usi.ucsd.edu/, and the mzspec values of the selected features and the metabolites recorded in the MS/MS databases were compared (Fig. S1). Annotations were performed according to the guidelines in ref. [36] (Table S2). Statistical analyses of metabolomic data were conducted in MetaboAnalyst v5.0, after filtering by the interquartile range (IQR) [37].

An automatic workflow for the analysis of the cell and supernatant fractions of *B. cinerea* and *B. subtilis* during coculture and *B. cinerea* treatment with TasA and fengycin at 6, 24 and 48 h (MSV000089552) can be accessed at https://massive.ucsd.edu/ProteoSAFe/dataset.jsp?task=b7579a1d14df42a09d30bd1418852db2 (feature-based molecular networking of the cell fraction:https://gnps.ucsd.edu/ProteoSAFe/status.jsp?task=4e4ade42ae60484886df1295747c5c71; feature-based molecular networking of the supernatant fraction: https://gnps.ucsd.edu/ProteoSAFe/status.jsp?task=c03281452dd342ccaf6962a051debb40).

### Native metabolomics

Native metabolomics experiments were conducted using the same chromatographic conditions as above. Post-column, 150 μl/min ammonium acetate buffer was introduced via a makeup pump and PEEKT splitter, while TasA protein (3 μm) was infused at 2 μl/min through the integrated syringe pump. The ESI settings were as follows: sheath gas flow, 40 arbitrary units; auxiliary gas flow, 10 arbitrary units; and sweep gas flow, 0 arbitrary units he auxiliary gas temperature was 150°C, spray voltage 3 kV, and inlet capillary temperature 253°C. The S-lens voltage was adjusted to 30 V. The MS scan range was set to 2500–4000 m/z with a resolution R_m/z_ 200 of 140 000 to 120 000 with 2 micro scans. MS acquisition was performed in all-ion fragmentation (AIF) mode with R_m/z_ 200 with 20% HCD collision energy and an isolation window of 2500–4000 m/z. For native LC–MS data analysis, raw ion spectra were analyzed with Xcalibur software (Thermo Scientific) to identify changes in protein mass. When a potential ligand was detected, retention times from the intact protein mass and metabolomics datasets were matched with an m/z offset corresponding to bacillaene B and dihydrobacillaene B.

### Minimum inhibitory concentration assays

Minimum inhibitory concentration (MIC) assays were conducted in liquid LB medium using the twofold serial dilution method as outlined by the guidelines of the Clinical and Laboratory Standards Institute (2003). The highest concentration tested for the compound SDA was 1000 μg/ml. All experiments were performed in triplicate, and the MIC was determined as the lowest antibiotic concentration that inhibited growth by >90%.

### Polysaccharide affinity assay

These assays were conducted following established protocols [38, 39], employing 30 μg/ml purified TasA. In brief, TasA monomers were incubated with 3 mg of chitin beads (New England Biolabs), crab shell chitin, chitosan, β-glucan, cellulose (all from Sigma) or xylan (TCI America) in 800 ml of water. Following gentle agitation at 4°C overnight, the insoluble fraction was pelleted by centrifugation (13 000 × g for 5 min), and the supernatant was collected. The insoluble fraction was washed three times with water and subsequently boiled in 1% SDS solution. The presence of protein in both the supernatant and pellet fractions was assessed via Tricine SDS–PAGE followed by Coomassie Brilliant Blue staining.

### Bacillaene extraction and purification

The isolation of bacillaene was performed with modifications to previously published protocols [40, 41]. In brief, cultures of *B. subtilis* (or ∆pks as a control) were grown in 1 L batches in LB broth and incubated overnight at 28°C with shaking. Following incubation, the cells were removed by centrifugation (9000 rpm, 10 min, 16°C), and the resulting cell-free supernatant was retained. Next, 250 ml of ethyl acetate was added and the mixture was incubated with agitation for 2 h, followed by 30 min of phase separation. The organic phase containing bacillaene was recovered and evaporated by lyophilization. The dried residue was resuspended in methanol and filtered through 0.45 μm filters (Econofltr PTFE). The extracts were separated using a preparative HPLC column (Eclipse XDB-C18 5 μm, 9.4 × 250 mm). The mobile phases consisted of 20 mM NaPi (A) and acetonitrile (B), with the following elution profile: 0–2 min at 35% B, 2–8 min from 35–40% B, 8–10 min at 40% B, 10–12 min from 40–35% B, and 12–15 min at 35% B. The flow rate of the mobile phase was set to 1 ml/min, and the absorbance was monitored at 362 nm using a photodiode array (PDA). The presence of bacillaene was confirmed by comparing the chromatograms of the wild-type extract with those of the ∆pks mutant extract, which lacked bacillaene, and by HPLC-MS by the MEDINA Foundation. The samples were analyzed using an Agilent 1200 Rapid Resolution HPLC connected to a Bruker maXis mass spectrometer. Separation was carried out using a Zorbax SB-C8 column (2.1 × 30 mm, 3.5 μm). The mobile phase consisted of two solvents: a 90:10 aqueous solution (solvent A) and a 10:90 aqueous solution (solvent B) of 13 mM ammonium formate and 0.01% TFA. The mass spectrometer was operated in positive electrospray ionization (ESI) mode, with the following settings: capillary voltage 4 kV, drying gas flow 11 L/min at 200°C, and nebulizer pressure 2.8 bar. Calibration was performed prior to injection using the TFA–Na ion cluster, and each sample was recalibrated by infusion of the TFA–Na calibrant before the chromatographic front. Each chromatographic run was processed using Bruker’s internal algorithm for component extraction, and the most intense peaks, both by TIC in positive mode and absorbance at 210 nm, were considered for accurate mass interpretation and molecular formula determination. Retention time and exact mass were then used as criteria to query the high-resolution MS database of the MEDINA Foundation.

### Docking

Automated tertiary structure modeling of the elongation factor (EF) protein from *B. cinerea* B05.10 (Acc. no. XP_001560460.1), derived from the Bchbs1 and Bcmef1 genes, were generated via AlphaFold [42]. To identify potential binding sites for bacillaene (PubChem ID: 25144999) on the EF proteins, we utilized the web-based SwissDock program [www.swissdock.ch/docking] [43] for automated molecular docking and thermodynamic analysis. SwissDock employs the EADock DSS algorithm to predict molecular interactions between a target protein and a small molecule. Docking was carried out using the “Accurate” setting with default parameters and no predefined region of interest (blind docking). Binding energies were calculated using CHARMM (Chemistry at HARvard Macromolecular Mechanics), integrated within SwissDock software, and the most favorable energies were assessed via fast analytical continuum treatment of solvation. The energy results were then scored and ranked on the basis of full fitness (kcal mol − 1), with spontaneous binding indicated by the estimated Gibbs free energy ΔG (kcal mol − 1). Negative ΔG values were interpreted as evidence of spontaneous binding. Structural visualization and analysis of docking results were performed using UCSF Chimera v1.8.

### Flow cytometry assays

Cells were cultured in 24-well plates containing 1 ml of MSgg medium at 28°C. At the indicated time points, the pellicle biofilm was removed, and the planktonic cells from the spent medium were collected by centrifugation (14 000 rpm, 3 min) and resuspended in 500 μl of PBS using a 25G needle. The cells were gently sonicated (12 pulses of 5 s and 30% amplitude) to ensure complete resuspension, fixed in 4% paraformaldehyde in PBS and washed three times in PBS. The flow cytometry runs were performed with 200 μl cell suspensions in 800 μl of GTE buffer (50 mM glucose, 10 mM EDTA, 20 mM Tris–HCl; pH 8), and the cells were quantified on a Beckman Coulter Gallios flow cytometer using 488 nm excitation. YFP fluorescence was detected with a 525/40 BP filter. The data were collected using Gallios Software v1.2 and analyzed with Flowing Software v2.5.1. Wild-type *B. subtilis* cells lacking the fluorescent reporter were used as a negative control for promoter activity measurements.

### Chitosan level quantification

Changes in the chitosan composition were determined via eosin Y labeling. The samples were subsequently resuspended in citrate–phosphate buffer (0.2 M NaH_2_PO_4_, 0.1 M K citrate; pH 6) with 1 μg/ml eosin Y (final concentration). After 10 min of incubation at room temperature, the cells were washed two times with citrate–phosphate buffer and placed on 1% agarose pads. Finally, the stained cells were imaged with an excitation wavelength of 488 nm, and emission was detected between 510 and 640 nm. Images were obtained using a Leica Stellaris 8 confocal microscope with a 63x NA 1.3 Plan APO oil-immersion objective. Processing and signal intensity measurement were performed via FIJI/ImageJ. For each experiment, the laser settings, scan speed, HyD detector gain and pinhole aperture were kept constant across all acquired images.

### Autophagosome staining and confocal microscopy

*Botrytis* spores were inoculated into 100 ml of potato dextrose broth (PDB) and incubated at 24–28°C under agitation (120 rpm) for 24 h to allow germination. Germinated spores were then treated with fengycin (10 μm) or rapamycin (1 μm) as a positive control for autophagy induction and incubated for 4 h under the same conditions. After treatment, samples were collected and stained with DAPGreen (Dojindo Laboratories) at a 1:1000 dilution for 30 min at room temperature in the dark. After staining, samples were washed once with PBS and immediately examined using a confocal laser scanning microscope equipped with a 488 nm excitation laser and appropriate emission filters. All images were acquired using identical acquisition settings for control and treated samples to enable direct comparison of fluorescence patterns and signal intensity.

## Results

### *Bacillus* extracurricular matrix mediates adhesion and bidirectional response in the interaction with *Botrytis*

**Figure 1:**
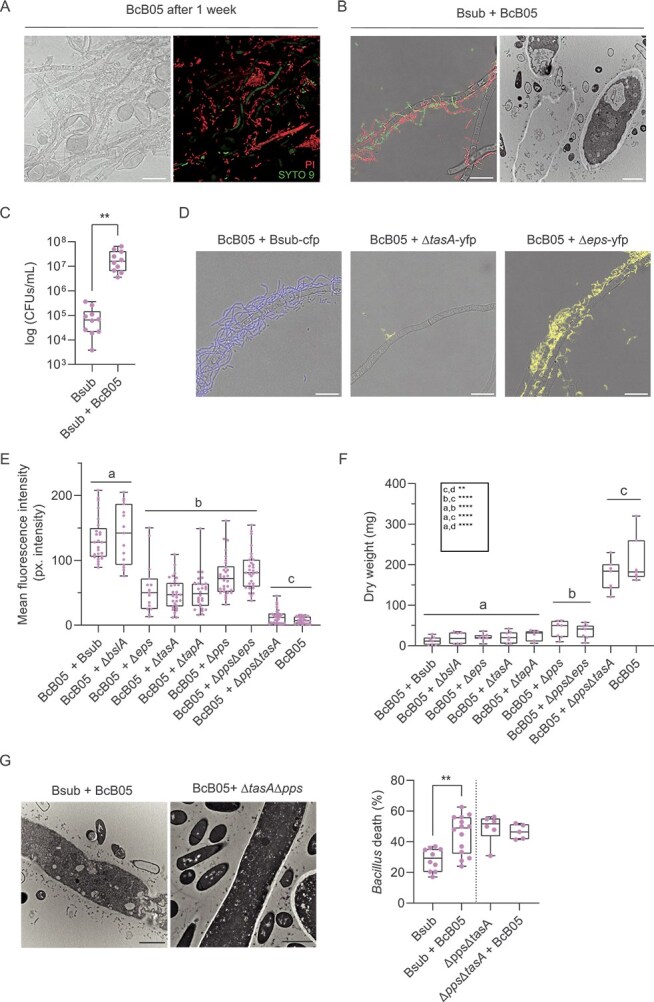
Bidirectional response and adaptive responses in the interaction between *B. subtilis* and *B. cinerea*. (A) Representative confocal microscopy images of *B. cinerea* after coculture with wild-type *B. subtilis* for 1 week. Left: bright-field image showing fungal and bacterial structures. Right: confocal microscopy image after live/dead staining with SYTO9 (green) and propidium iodide (PI, red). SYTO9 stains live bacterial cells and some fungal structures green, while PI stains dead cells red. (B) Left: confocal microscopy images showing *B. subtilis* mortality near *B. cinerea* hyphae after 24 h, visualized using live/dead staining, highlighting significant bacterial cell death near fungal structures, indicative of bidirectional response. Right: transmission electron microscopy images showing *B. cinerea* cell leakage and *B. subtilis* ghost cells surrounding fungal hyphae. (C) Quantification of the total *B. subtilis* population in coculture after 1 week, demonstrating an overall increase compared with that of the monoculture. (D) Representative images showing impaired adhesion of *B. subtilis* mutants lacking tasA to *B. cinerea* hyphae. (E) Quantification of ROS levels from confocal images of *B. cinerea* during coculture with wild-type *B. subtilis (*Bsub), mutants lacking structural and nonstructural ECM components (Δpps, ΔtasA, ΔtapA, Δeps, and ΔbslA) and double mutants (Δpps Δeps and ΔppsΔtasA) after 24 h of interaction. (F) Fungal growth of *B. cinerea* during coculture assays after 24 h of interaction. (G) Quantification of the percentage of *B. subtilis* cell death in monoculture and during coculture with *B. cinerea*, including comparisons with the ΔtasAΔpps mutant strain, showing the influence of these ECM components on fungus-induced bacterial mortality. Scale bars for confocal microscopy represent 20 μm, and scale bars for TEM images represent 2 μm. The whisker plot shows all the measurements (pink dots), medians (black line), and minimum and maximum values (whisker ends). Statistical analyses were performed on at least three biological replicates. Statistical significance was assessed using a t test, with two asterisks indicating significant differences at *P* < .01.

Microbial interactions are inherently dynamic and are shaped by evolving exchanges between organisms and their environment. This ecological fluidity is especially pronounced in antagonistic or competitive relationships within microbial communities, where organisms constantly adapt to maintain ecological balance or eliminate competitors [44–47]. Historically, the interaction between *B. subtilis* and *B. cinerea* has been described as unidirectionally antagonistic, driven by the ability of *B. subtilis* to suppress fungal growth through the secretion of antifungal compounds, including plipastatins and surfactins [48]. However, our observations revealed a more complex and dynamic interaction over time. In the short term, *Bacillus* inhibited *Botrytis* growth; however, some of the fungal population survived after one week and even one month of interaction (Fig. 1A), an observation that suggests the existence of a defense mechanism activated by *Botryti*s to persist in a chemically hostile environment. Moreover, rather than simply being the attacker, the *Bacillus* population also experiences significant mortality during this interaction, particularly the bacterial cells that are in close contact with fungal hyphae (Fig. 1B). These findings, indicative of bidirectional antagonism, challenge the conventional view of this interaction as strictly unidirectional and point toward a more balanced relationship wherein both organisms may employ adaptive defense mechanisms. Indeed, in the long term (1 week), the total *Bacillus* population was comparatively larger than that in *Bacillus* monoculture (Fig. 1C, Fig. S2A), despite the higher percent mortality of *Bacillus* cells attached to *Botrytis* hyphae. This result indicates that individual bacterial cells are more likely to die when they are in close contact with *Botrytis*, although the overall *Bacillus* population increases during the interaction, possibly due to a beneficial environmental niche or nutrient availability created by the presence of the fungus. This population expansion, however, is not attributable to sporulation, since *B. cinerea* does not trigger spore formation in *Bacillus* (Fig. S2B).

To investigate the mechanism underlying the dynamic interaction between *Bacillus* and *Botrytis*, we initially examined the transcriptomic profiles of both organisms after 6 h of coculture. Dual RNA-seq analyses revealed deregulation of key pathways involved in glutathione metabolism, secondary metabolite biosynthesis, or phospholipid metabolism in *Botrytis* (Fig. S2C). Specifically, we observed the overexpression of *Botrytis* genes related to the fungal matrix, microbody lumen, cell wall, peroxisomes, plasma membrane and peroxisomal matrix (Fig. S3A). These results suggest the activation of a defensive strategy to preserve structural integrity and manage putative cellular damage associated with oxidative stress. Moreover, we observed notable downregulation of genes associated with the cytoskeleton, further indicating potential reorganization or damage of the internal structures of *Botrytis* due to the interaction with *Bacillus* (Fig. S3B). In the interaction, genes involved in secondary metabolite production (Fig. S3C) and genes related to *Bacillus* ECM biosynthesis (TasA, TapA, EPSs, and BslA) were upregulated in the *Bacillus* population. These results, along with the relevance of the ECM in *Bacillus* ecology and communication with other organisms, led us to investigate the role of structural ECM components in the colonization of fungal hyphae and the antagonism toward *Botrytis*. Adhesion to hyphae was clearly impaired in mutants lacking *eps* or *tasA* (Fig. 1D). The removal of any ECM component, including TasA, TapA, or EPSs but not BslA, resulted in a reduction in reactive oxygen species (ROS) levels in *Botrytis* (Fig. 1E). However, no reduction in the mortality rate or fungal biomass of *Botrytis* during coculture with ΔtapA or ΔtasA strains was detected (Fig. 1F), likely because of the higher levels of fengycin production reported for the ΔtasA mutant [49]. The plipastatin produced by *B. subtilis* is classified in the fengycin family because of its similar structure [50]. Therefore, herein, we will refer to both as fengycin, including when discussing *Bacillus-*derived compounds. The lack of adhesion observed in the ΔtapA mutant, coupled with reduced ROS levels, was intuitively attributed to the inability of this mutant to efficiently expose TasA on the cell surface [11, 51]. Compared with *Bacillus* WT cells, the ∆pps mutant, unable to synthesize fengycin, triggered lower levels of ROS production in *Botrytis* (Fig. 1E). The ΔtasAΔpps double mutant strain lost practically all of its antifungal activity, which correlated with levels of ROS comparable to those in untreated *Botrytis* hyphae (Fig. 1E and F). Therefore, TasA and fengycin seem to synergistically influence the antagonistic activity of *Bacillus* cells toward *Botrytis*. This pattern was confirmed by the behavior of the ΔtasAΔpps double mutant, which exhibited high basal mortality even in monoculture, attributed to the absence of TasA [49], but showed no additional lethality upon exposure to *Botrytis*. Conversely, the WT strain displayed a marked increase in death rate during co-inoculation (Fig. 1G), consistent with a targeted fungal response that is specifically triggered by the presence of TasA and/or fengycin [49].

### Lysophosphatidylcholine and reactive oxygen species accumulation in *Botrytis* hyphae is correlated with the presence of TasA

**Figure 2:**
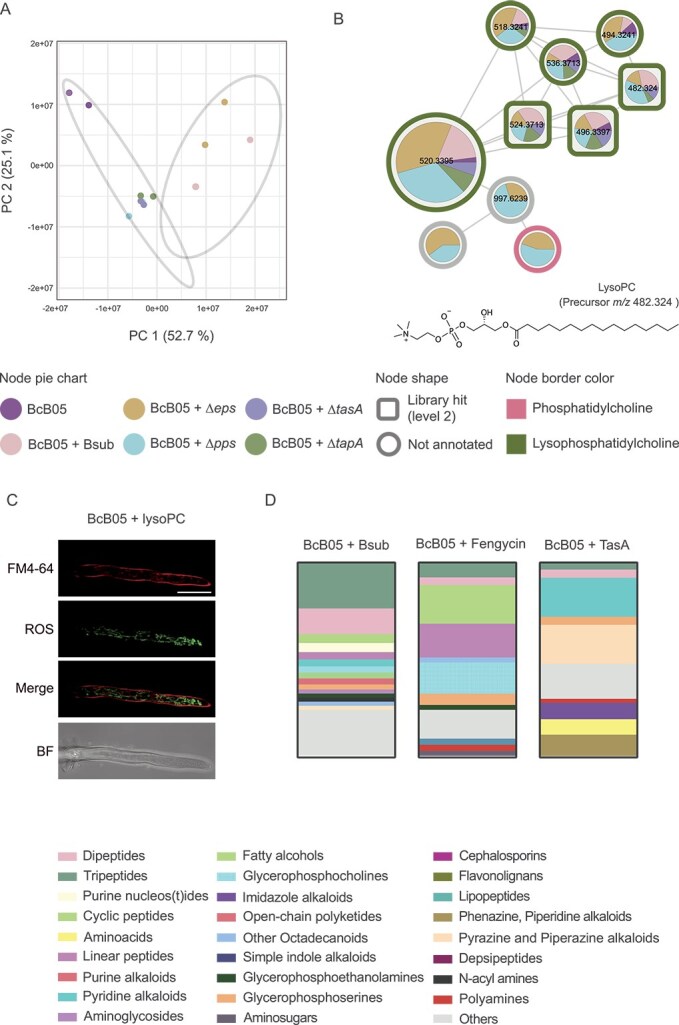
ECM structural components of *B. subtilis* drive lysophosphatidylcholine accumulation and oxidative stress in *B. cinerea*. (A) PCA 2D score plot of the metabolome of the *B. cinerea* cell fraction after 24 h of coculture with wild-type *B. subtilis* or ECM mutants (Δpps, ΔtasA, ΔtapA, and ΔEPS). *B. cinerea* Cocultured with the Δpps, ΔtasA, or ΔtapA mutant clustered closely with the *B. cinerea* monoculture control, whereas *B. cinerea* cocultured with wild-type *B. subtilis* and the Δeps mutant formed distinct clusters. The percentage of variation explained by each principal component is indicated on the axes. (B) Molecular family of features differentially abundant in the cell fraction of *B. cinerea* after 48 h of treatment with wild-type *B. subtilis* or ECM mutants (Δpps, ΔtasA, ΔtapA, and ΔEPS), annotated as lysophosphatidylcholines according to NPClassifier. The chemical structures of annotated features and their average masses on the basis of spectral matches to GNPS libraries are also represented for the corresponding molecular families. Pie charts indicate the peak abundance of each metabolite under the corresponding conditions. The node shape indicates the level of identification according to a previous publication [36]. Lyso-PC accumulation is significantly reduced in interactions with ΔtasA or ΔtapA mutants, suggesting a dependence on ECM structural components. (C) ROS levels in *B. cinerea* cultures treated with 150 μm commercial lyso-PC, confirming its role in triggering oxidative stress. (D) Stacked bar plots represent the relative abundance of chemical classes for the top 250 features significantly increased in the *B. cinerea* cell fraction after 24 h of treatment with TasA, 10 μm fengycin, or wild-type *B. subtilis*, compared with untreated *B. cinerea*. The features were ranked using volcano plots generated in MetaboAnalyst. Metabolite chemical class prediction was performed using SIRIUS, and the metabolites were classified with NPClassifier. Scale bars represent 20 μm.

Considering the relevance of the ECM in the antagonistic interaction of *Bacillus* and *Botrytis*, and the differences in *Bacillus* mortality on the basis of the presence of certain ECM components, we aimed to elucidate potential metabolic changes that might modulate the interaction between these two microorganisms. We conducted a time-course comparative analysis of *Bacillus* strains (WT and derivative ECM mutants), and *Botrytis* metabolomes in monocultures and pairwise interactions at 6, 24, and 48 h. Non-targeted metabolomics analysis revealed that multiple metabolites, including glycerophosphocholines, glycerophosphoethanolamines, cyclic lipopeptides, dipeptides, tripeptides, and alkaloids, were differentially accumulated during the interaction (Fig. S4A). *Bacillus* notably presented elevated levels of cyclic lipopeptides and glycerophosphoethanolamines, which are known for their potent antifungal properties (cyclic lipopeptides), and their involvement in the regulation of membrane stress responses (glycerophosphoethanolamines), respectively, suggesting a coordinated chemical strategy by *Bacillus* to destabilize fungal membranes and trigger cellular stress [7, 52, 53]. In contrast, changes in glycerophosphocholine levels in *Botrytis* indicated active membrane remodeling in response to *Bacillus*, potentially aiding in adaptation to environmental stresses [54–58]. Additionally, the accumulation of dipeptides, tripeptides, and alkaloids, which are typically associated with stress and intercellular signaling, suggested a reorganization of metabolic pathways, potentially influencing the growth and survival of both organisms (Fig. S4A and B). Principal component analysis of the cell fraction after 24 h of interaction revealed distinct clustering patterns between *Botrytis* treated with the *Bacillus* WT or single ECM mutants. Consistent with the proposed prominent role of TasA, and fengycin in the antagonistic activity, the metabolomes of *Botrytis* cocultured with the ∆pps, ∆tasA, or ∆tapA mutants clustered closely with those of *Botrytis* grown in monoculture, whereas the *Bacillus* WT and mutant lacking EPSs formed a separate cluster (Fig. 2A). These results suggest that the interaction with *Bacillus* WT or the ∆eps mutant induces substantial shifts in the *B. cinerea* metabolome. In contrast, the metabolome of *Botrytis* treated with the ΔtapA, ΔtasA, or Δpps mutant showed minimal changes and closely resembled that observed without treatment, indicating the critical contribution of TasA, and fengycin in driving the metabolic modulation of *Botrytis* during the interaction.

Further analysis of the metabolomes revealed accumulation of lysophosphatidylcholine (lyso-PC) in the *Botrytis* cell fraction after coculture with *Bacillus*. Lyso-PC is a lipid signaling molecule associated with ROS generation, cellular damage, and inflammatory processes in other biological systems [58–63]. Lyso-PC was practically absent in *Botrytis* treated with the ∆tasA or ∆tapA mutant, a finding that led us to correlate the accumulation of lyso-PC with the presence of *Bacillus* ECM components (Fig. 2B). We hypothesize that the accumulation of lyso-PC might correlate with the levels of ROS accumulated in *Botrytis* during the interaction (Fig. 1E). Commercial lyso-PC exogenously added to *Botrytis* cultures triggered significant increase in ROS levels (Fig. 2C, Fig. S5A), suggesting that lyso-PC produced by *B. cinerea* would be a molecule contributing to the oxidative stress observed during the *Bacillus-Botrytis* interaction, a physiological response triggered by the presence of the *Bacillus* ECM structural component TasA. However, the level of ROS produced after the addition of lyso-PC did not appear to lead to fungal death or growth inhibition (Fig. S5B), suggesting that lyso-PC may function primarily in signaling rather than inhibiting the growth of *Botrytis*. According to this finding, the external addition of purified TasA to *Botrytis* reproduced the accumulation of ROS observed in previous coculture experiments and promoted the accumulation of lyso-PC in *Botrytis* cells. Fengycin also triggered ROS accumulation in *Botrytis* hyphae; however, the level of lyso-PC accumulation did not increase significantly.

To better understand the specific metabolic changes triggered by the most active ECM components, we analyzed the metabolome of *B. cinerea* treated with purified TasA or fengycin. Chemical class analysis of non-targeted metabolomics data with CANOPUS [33] revealed that during the interaction of *Botrytis* with *Bacillus*, glycerophosphocholines, dipeptides, tripeptides, cyclic peptides, and alkaloids accumulated after 24 h, as shown in Fig. S4B. Fengycin-treated *Botrytis* exhibited accumulation of dipeptides, tripeptides, and glycerophosphocholines, a pattern consistent with membrane disruption, as these small amphipathic molecules may integrate into lipid bilayers, compromise membrane integrity, and promote leakage of intracellular contents, ultimately leading to cell death [64, 65]. Similarly, TasA-treated *Botrytis* presented minor accumulation of dipeptides, and tripeptides compared with fengycin-treated *Botrytis*, as well as pronounced accumulation of alkaloids, particularly piperazine, and pyridine alkaloids (Fig. 2D). These findings suggest that dipeptides, and tripeptides accumulate in the presence of both ECM components. Glycerophosphocholine production is driven by fengycin, and alkaloid production is associated with TasA. This differential response of *Botrytis* at the metabolic level led us to investigate specific physiological and anatomical damage induced by these molecules produced by *Bacillus*.

### TasA modifies structural integrity of *Botrytis* hyphae, compromising virulence

**Figure 3:**
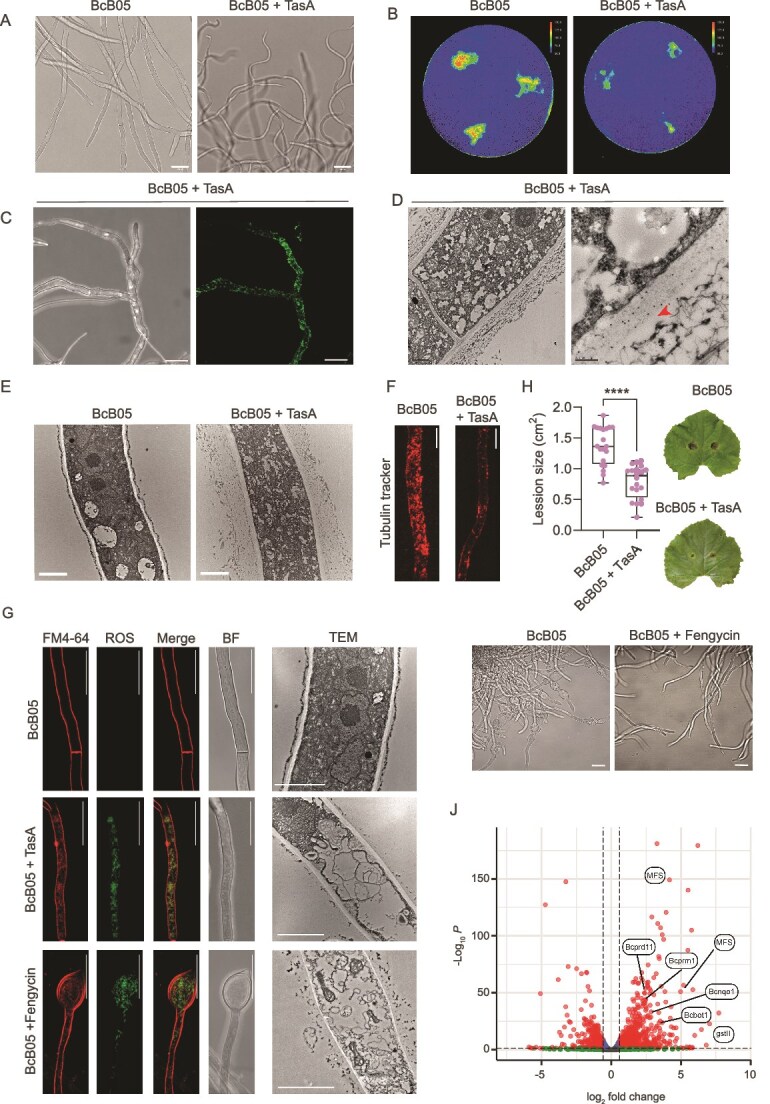
TasA and fengycin differentially impact *B. cinerea* physiology. (A) Macroscopic phenotypic analysis showing the curling morphology of *B. cinerea* hyphae upon treatment with purified TasA, likely due to disorganization of the fungal cell surface. Scale bars represent 20 μm. (B) Magnetic resonance imaging-based T2 relaxation time analysis showing that untreated *B. cinerea* macrocolonies retain water heterogeneously, with higher T2 times at the colony center. In contrast, TasA-treated colonies presented a uniform distribution of water content and relatively low T2 relaxation times, suggesting disruption of fungal matrix integrity and reduced water retention. (C) Immunocytochemistry using anti-TasA antibodies demonstrating extensive decoration of fungal hyphae after *B. cinerea* treatment with 3 μm purified TasA. Scale bars represent 20 μm. (D) Transmission electron micrograph of negatively stained thin sections of *Botrytis* hyphae with immunogold labeling revealing TasA accumulation in the outer and inner layers of the fungal cell wall, which is primarily composed of chitin and chitosan. Signals corresponding to immunogold particles are indicated with red arrowheads. The inner cell wall (IC) and outer cell wall (OC) are annotated in the image for clarity. The scale bar represents 1 μm for the image on the left and 0.2 μm for the zoomed-in image on the right. (E) TEM images of untreated hyphae display a sharply defined β-glucan layer outside the cell wall (left), whereas TasA-treated hyphae exhibit disorganized β-glucan structures (right). The IC and OC are annotated in the image for clarity. The scale bar represents 2 μm. (F) Tubulin staining (tubulin tracker deep red, Thermo T34077) of *B. cinerea* hyphae revealed significantly fewer tubulin foci in TasA-treated samples than in control samples, indicating cytoskeletal disorganization. (G) Confocal microscopy images of ROS levels in 3 μm TasA-treated *B. cinerea* hyphae, obtained using double staining of DHR123 with FM4–64 to stain the membrane, and TEM images showing ultrastructural damage, including autophagosome formation, in TasA-treated *Botrytis*. Scale bars: 20 μm for confocal microscopy images and 2 μm for TEM images. (H) *In planta* experiments showing reduced lesion size and fungal colonization in plants treated with TasA compared with those in untreated controls. The whisker plot shows all the measurements (pink dots), medians (black line), and minimum and maximum values (whisker ends). For all experiments, the results from at least three biological replicates are shown. Statistical significance was assessed via a t test, with quadruple asterisks indicating significant differences at *P* < .0001. (I) Brightfield microscopy of *B. cinerea* hyphae demonstrating extensive chlamydospore formation upon fengycin treatment (lower panel) compared with the control treated with methanol (upper panel). Scale bars equal 20 μm. (J) Volcano plot of DEGs identified by RNA-seq in *B. cinerea* treated with fengycin for 6 h and untreated hyphae. *P*-values were calculated on the basis of the Fisher method using nominal *P*-values provided by edgeR and DEseq2. The dashed lines represent the thresholds defined for *P* (horizontal) and the fold change (vertical) for a gene to be considered a DEG. genes related to virulence and detoxification pathways are labeled as follows: MFS, gstII (glutathione S-transferase), Bcprd11 (peroxidase, involved in the response to oxidative stress), Bcprm1 (serine peptidase, involved in fungal survival), Bcnqo1 (oxidoreductase) and Bcbot1 (botrydial biosynthesis gene, involved in secondary metabolism).

*Botrytis* accumulates ROS in response to the presence of *Bacillus* ECM, however, evident inhibition of fungal growth or accumulation of lyso-PC was observed in the presence of fengycin or TasA, respectively. The addition of purified TasA to *Botrytis* cultures promoted a curling morphology of hyphae (Fig. 3A), a macroscopic phenotype reasonably associated with alterations of the fungal cell surface [66, 67]. To examine the hypothetical impact of TasA on the biophysical properties of the fungal matrix, we conducted MRI analyses to assess T2 relaxation times across different colony regions, which reflect water content and diffusion. High-resolution images of untreated *Botrytis* macrocolonies revealed significant heterogeneity, with varying water contents and relatively high T2 relaxation times in the center of the colonies. In contrast, *Botrytis* treated with TasA presented a homogeneous distribution, with uniformly lower T2 times. This finding suggested the existence of an intact matrix in *Botrytis* colonies that is able to retain water within the colony, and that disruption of matrix integrity in the presence of TasA reduces water retention, and thus, structural organization (Fig. 3B, Fig S6A).

The relevance of TasA for the cellular attachment of *Bacillus* cells to *Botrytis* hyphae led us to initially evaluate the role of this protein in the disorganization of the fungal cell wall. Immunocytochemistry analysis using anti-TasA antibodies revealed that the fungal hyphae were extensively decorated with the fluorescent signal associated with TasA (Fig. 3C). Consistently, immunofluorescence analysis of *Bacillus*–*Botrytis* co-cultures confirmed the localization of TasA on the fungal surface (Fig. S6B), reinforcing its direct involvement in the interspecies interface. Transmission electron microscopy (TEM) analysis of thin sections of *Botrytis* hyphae and immunogold labeling confirmed the accumulation of TasA-related signals in the outer and inner fungal cell wall layers, which contained mainly chitin and chitosan, the two major components of the fungal cell wall (Fig. 3D). TEM control images of *B. cinerea* are shown in Fig. S6C. The affinity of TasA for both chitin and chitosan was biochemically demonstrated via polysaccharide affinity assays (Fig. S7A). In the TEM analysis, however, any alteration in the chitin-chitosan layer was disregarded, and noticeable disorganization of the β-glucan layer of the fungal cell wall was observed following treatment with TasA, which, in untreated hyphae, appeared as a sharply defined electrodense line outside the less electrodense chitin-chitosan layer (Fig. 3E).

Fungal shape and structural integrity are largely regulated by the cytoskeletal element actin filaments and microtubules [68], and disruption of the cytoskeleton may induce a curling morphology. Therefore, we reasoned that disorganization of the β-glucan layer induced by TasA might cause cytoskeleton dysfunction, weaken the structural integrity of the fungal cell wall and compromising the ability of *Botrytis* to maintain its typical hyphal architecture and function. Diverse lines of experimental evidence support this hypothesis: (i) downregulation of genes related to cytoskeletal organization in transcriptomic analysis of TasA-treated *Botrytis* hyphae (Fig. S7B), (ii) a significant decrease in tubulin foci of hyphae of *Botrytis* treated with TasA upon specific staining of the cytoskeleton with a tubulin tracker (Fig. 3F, Fig. S7C), and (iii) cytoplasmic damage characterized by autophagosome formation observed via TEM analysis of thin sections of *Botrytis* hyphae (Fig. 3G). Collectively, these findings demonstrate that in addition to facilitating the physical contact of *Bacillus* cells with *Botrytis* hyphae, TasA disrupts the physical and molecular architecture of *Botrytis* cells. These results confirm the active participation of TasA in the arsenal of molecules secreted by *Bacillus* to efficiently antagonize *Botrytis.* The failure of EPSs to induce significant changes at the ultrastructural level (Fig. S8A) is likely related to the limited access of this bacterial polymer to the fungal cell wall or membrane. However, the increase in ROS levels suggested that *Botrytis* can sense the presence of EPS, most likely triggering a differential response to TasA (Fig. S8B). We further demonstrated in an in vivo plant model (melon) that the structural disorganization and morphological changes triggered by TasA translated into a reduced ability of *Botrytis* to induce symptoms in leaves (Fig. 3H). Microscopic analyses of infected tissues revealed that TasA treatment directly altered fungal morphology, inducing thickened, swollen hyphae with irregular branching and limited penetration into host tissues (Fig. S8C). While these changes are consistent with a direct antifungal effect, they do not exclude a potential role for TasA in priming or enhancing plant defense responses.

Fengycin is known to target fungal membranes, and consistent with this, the cytoplasmic disorganization of *Botrytis* hyphae was more pronounced than that induced by TasA. The most noticeable cytological alterations were autophagosome formation (Fig. S8D), plasmolysis (Fig. 3G), and extensive formation of chlamydospores (Fig. 3I), which are differentiated fungal cells associated with resistance to external aggressions [69–71]. To confirm that these membranous structures correspond to autophagosomes, *B. cinerea* hyphae were stained with the autophagosome-specific probe DAPGreen and analyzed by confocal microscopy. Fengycin-treated samples displayed numerous discrete fluorescent puncta, comparable to those observed in the rapamycin-treated positive control (Fig. S8E), confirming that fengycin induces autophagosome formation. In line with these pronounced cytological rearrangements, transcriptomic analysis of *Botrytis* exposed with fengycin after 6 h revealed upregulation of virulence-related genes and detoxification pathways, particularly those associated with glutathione, oxidoreductases, and major facilitator superfamily (MFS) transporters. These findings indicate the activation of a chemical defense mechanism oriented toward mitigating the oxidative stress and cytological damage inflicted by fengycin (Fig. 3J).

### Antibacterial oxylipins differentially produced by *Botrytis* in response to TasA or fengycin

**Figure 4:**
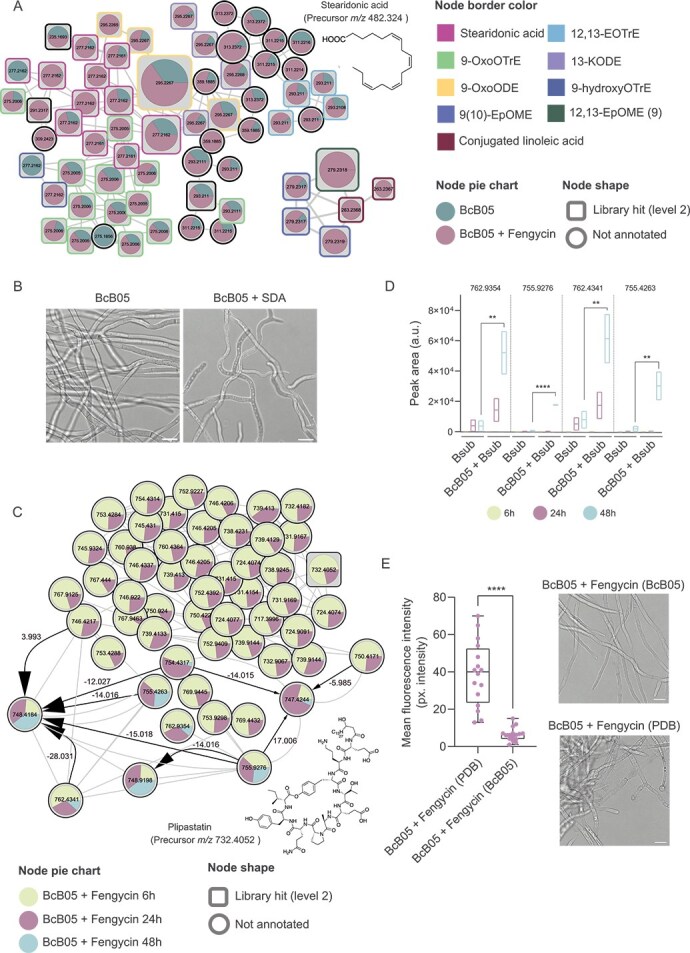
Counteracting *B. subtilis* aggression: Oxylipin production and fengycin enzymatic degradation. (A) Molecular families of features differentially abundant in the supernatant fraction of *B. cinerea* after 24 h of treatment with 10 μm fengycin or no treatment. The chemical structures of annotated features and their average masses on the basis of spectral matches to GNPS libraries are also represented for the corresponding molecular families. Pie charts indicate the peak abundance of each metabolite under the corresponding conditions. The node shape indicates the level of identification according to a previous publication [36]. The node border color indicates the compound name according to the GNPS library. (B) Representative confocal microscopy images of untreated *B. cinerea* (left) or *B. cinerea* treated with 1 μg/ml commercial SDA (right), triggering hyperbranching. Scale bars equal 10 μm. (C) Molecular family analysis of fengycin and its structural variants in *B. cinerea* supernatants after the addition of 10 μm fengycin, which were sampled at 6, 24, and 48 h. pie charts represent the mean peak abundance of metabolites at each time point, with node shapes indicating the level of metabolite identification. The arrows indicate the chemical directionality of the modifications observed, with arrow sizes corresponding to ChemProp scores. The labels above the arrows reflect the mass differences between related metabolites. (D) Floating bar plots showing the peak abundances of four selected putative degradation products of fengycin in *B. subtilis* cultures growing alone and in coculture with *B. cinerea* at 6, 24, and 48 h. (E) Left: quantification of ROS levels in *B. cinerea* after treatment with fengycin preincubated in PDB medium for 24 h versus fengycin preincubated with *Botrytis* for the same amount of time. Lower ROS levels were detected in *B. cinerea* treated with fengycin preincubated with *B. cinerea*, suggesting neutralization of fengycin activity. The whisker plot shows all the measurements (pink dots), medians (black line), and minimum and maximum values (whisker ends). For all experiments, the results from at least three biological replicates are shown. Statistical significance was assessed via a t test, with quadruple asterisks indicating significant differences at *P* < .0001. Right: confocal microscopy images showing cytological damage and chlamydospore formation in *B. cinerea* treated with fengycin preincubated with PDB as a control, whereas *B. cinerea* treated with fengycin preincubated with *B. cinerea* presented no apparent cytological damage. Scale bars equal 20 μm.

We determined how *Botrytis* hyphae (cell fraction, Fig. 2D, Fig. 3G) respond differentially metabolically and anatomically to TasA or fengycin, two active *Bacillus* ECM components involved in the interaction. However, the factors delivered by *B. cinerea* that are lethal to *B. subtilis* cells (Fig. 1B) remain elusive. To illuminate the change in metabolite production associated with hyphal damage in *B. cinerea*, we performed non-targeted metabolomics analyses of the *Botrytis* supernatant at different time points (6, 24, and 48 h) after treatment with purified TasA or commercial fengycin. Consistent with the morphological changes (Fig. 3G), our data revealed that TasA and fengycin differentially impacted *Botrytis* physiology, the metabolome and most likely the fungal lifestyle. Fengycin treatment led to substantial secretion of metabolites by *Botrytis* into the media, particularly after 24 h, and TasA-treated *Botrytis* reduced the overall accumulation of metabolites (Fig. S9). The pool of accumulated metabolites in the fungal supernatant fraction under both treatments included oxygenated fatty acids or oxylipins, especially after treatment with fengycin (Fig. 4A). Oxylipins are derived from linoleic and linolenic acids, and are known as developmental regulators or mediators of interspecies communication across various biological systems, including mammals, bacteria, fungi, and plants [72–74]. In filamentous fungi*,* oxylipins constitute an essential defensive mechanism for fungal adaptation to the environment [75–77]. The linolenic-derived oxylipins detected included stearidonic acid (SDA), 9-OxoOTrE, trans-EKODE, 13-OxoODE and 9S-HOTrE, and the linoleic-derived epoxides detected included 12,13-EpOME and 9(10)-EpOME (Fig. S10A). One of the most abundant oxylipins, SDA, exhibited potent antibacterial effects, significantly contributing to *B. subtilis* mortality at concentrations as low as 6.25 μg/ml (Fig. S10B). This finding endorses SDA as a potential key factor in the defensive arsenal of *Botrytis* activated upon the differential cellular aggression inflicted by the *Bacillus* ECM molecule fengycin and, to a lesser extent, TasA.

In addition to their antibacterial activity, fungus-derived oxylipins seem to mediate intrapopulation communication within fungi [73]. Accordingly, SDA exogenously added to *Botrytis* culture induced hyphal branching [78] (Fig. 4B, Fig S10C). Hyperbranching in filamentous fungi is perceived as an ecological strategy that enhances the ability of fungi to explore and capture nutrients, as well as provides protection against threats [78]. This behavior is part of an interference competition strategy that increases the likelihood of survival and fitness in resource-limited environments [79]. Therefore, the production of these oxylipins by *Botrytis* seems to exemplify a dual function: damaging competing microbes such as *Bacillus* while promoting alternative adaptive growth strategies.

### *Botrytis* enzymatically degrades fengycin, neutralizing its antifungal activity

Two alternative strategies could explain the long-term resistance of *Botrytis* to the physicochemical effects of *Bacillus*: (i) antagonism toward *Bacillus* cells (Fig. 1B) and (ii) neutralization of the toxicity of the antifungal molecules produced by *Bacillus*. We have proposed oxylipins as one type of antibacterial agents secreted by *Botrytis* in response to the action of fengycin (Fig. 4A), with a deleterious effect on *Bacillus* cells, thus, we focused on demonstrating the implications of the second strategy. Considering the relevance of fengycin in the antifungal activity of *B. subtilis*, we analyzed the degradation of this lipopeptide during the interaction. By leveraging non-targeted metabolomics data from serial incubation with fengycin, we analyzed proportionality changes (anticorrelation behavior) in chemically related metabolites through molecular networking and the ChemProp2 computational tool [80]. This analysis revealed a significant reduction in fengycin levels in the cell-free supernatant 24 to 48 h after the addition of commercial fengycin to *Botrytis* cultures. This reduction in the original fengycin level was accompanied by the accumulation of specific structural variants of fengycin with *m/z* values of 762.9354, 755.9276, 762.4341, and 755.4263, likely representing degradation products (Fig. 4C). Similar dynamics were observed for native fengycin produced by *Bacillus* upon interaction with *Botrytis*, indicating the ability of *Botrytis* to chemically modify fengycin (Fig. 4D). To evaluate the biological implications of this degradation, *Botrytis* was incubated with commercial fengycin for 24 h, after which the supernatant was added to fresh *Botrytis* cells. Fengycin exposed to *Botrytis* lost its antifungal activity, as demonstrated by the absence of fungal growth inhibition, lack of cellular damage, and lack of increase in ROS levels, compared with that of control fengycin incubated in PDB simultaneously (Fig. 4E). Two complementary observations supported the active enzymatic degradation of fengycin carried out by *Botrytis* during the interaction: (i) the fact that the pH of the culture did not change significantly, and (ii) the specific fengycin degradation products with known enzymatic degradation profiles reported in the literature [81]. Additional MS/MS analyses using optimized collision energies revealed that the degraded fengycin variants underwent oxidative and methylation-type modifications, as well as potential partial linearization of the peptide backbone (Fig. S11). These chemical alterations are consistent with known enzymatic degradation patterns, supporting the notion that *B. cinerea* actively modifies fengycin to neutralize its antifungal activity. To identify putative enzymes involved in the chemical transformation of fengycin, we analyzed the genes that were upregulated in *Botrytis* following fengycin treatment or upon interaction with wild-type *Bacillus* via a previously described workflow involving RNA-Seq data [65]. We focused on proteins predicted to be extracellular and conducted a search for conserved protein domains to uncover key functional motifs involved in their activity, identifying putative enzymes involved in fengycin degradation (Table S3). This is a notable observation since the degradation of lipopeptides is reported to occur during competition with other bacteria [82] but not fungi. This finding indicates that there could be an additional defensive mechanism used by *Botrytis* and probably other filamentous fungi to neutralize antifungal molecules produced by *Bacillus*.

### Degradation of fengycin coincides with the secretion of fungistatic bacillaene by *Bacillus*

**Figure 5:**
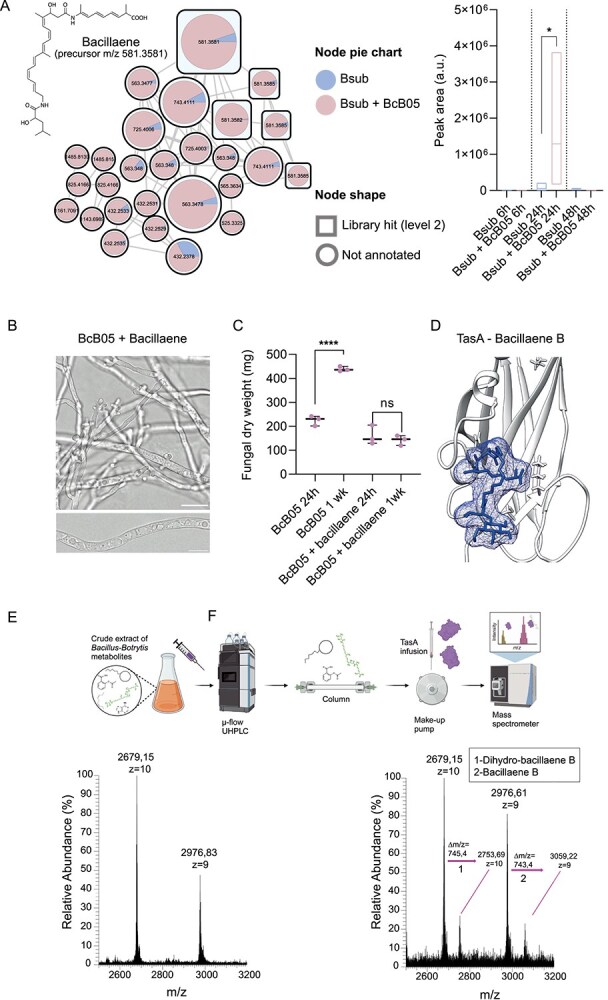
Interaction with *B. cinerea* triggers bacillaene production, revealing its fungistatic effects. (A) Left: molecular family analysis of bacillaene in *B. cinerea* supernatant after interaction with *B. subtilis* compared with that in the *B. subtilis* monoculture at 24 h. pie charts represent the mean peak abundance of metabolites in the supernatant, with node shapes indicating the level of metabolite identification according to GNPS libraries. Right: Floating bar plots showing the peak abundance of bacillaene (precursor m/z 581.3581) in *B. subtilis* supernatants from monoculture and coculture with *B. cinerea* at 6, 24, and 48 h, highlighting bacillaene accumulation during interactions at 24 h. statistical significance was assessed via a t test, with single asterisks indicating significant differences at *P* < .05. (B) Confocal microscopy images of *B. cinerea* treated with 60 μg/ml purified bacillaene for 24 h, showing cytoplasmic vacuolization and hyphal tip splitting. Scale bars represent 20 μm. (C) Quantification of the fungal mass after 24 h and 1 week demonstrated halted fungal growth upon bacillaene treatment, confirming its fungistatic activity. The whisker plot shows all the measurements (pink dots), medians (black line), and minimum and maximum values (whisker ends). For all experiments, the results from at least three biological replicates are shown. Statistical significance was assessed via a t test, with quadruple asterisks indicating significant differences at *P* < .0001. (D) Molecular docking analysis predicted a strong binding interaction between bacillaene B (precursor m/z 743.4) and TasA. (E) Top: overview of the native metabolomics workflow analyses between purified TasA and the crude extract of metabolites. Left: mass spectrum of TasA alone. Right: mass spectrum of TasA in the presence of the crude metabolite extract from the *Bacillus-Botrytis* coculture, revealing a distinct mass shift corresponding to the formation of the TasA-bacillaene complex when bacillaene passed through the column. This shift indicates the binding of bacillaene a, an isoform of bacillaene functionalized with a hexose group.

Following the degradation of *Bacillus* primary antifungal compounds with activity against *Botrytis*, metabolomic analyses revealed an increase in the production and accumulation of bacillaene, another secondary metabolite from the *Bacillus* chemical arsenal [83]. Specifically, bacillaene accumulated at significantly greater levels in the supernatants of *Bacillus* cells cocultured with *Botrytis* than in those of *Bacillus* cells grown in monoculture after 24 h (Fig. 5A). Non-targeted metabolomic analysis of the interaction of *Botrytis* with the ∆pps mutant (unable to produce fengycin) revealed elevated accumulation of bacillaene compared with the levels accumulated during the interaction with wild-type *Bacillus*, indicating compensatory regulation between fengycin and bacillaene (Fig. S12A). This finding indicates a flexible defensive strategy adopted by *Bacillus* that involves the use of bacillaene in response to the fengycin-neutralizing activity of *Botrytis*.

**Figure 6:**
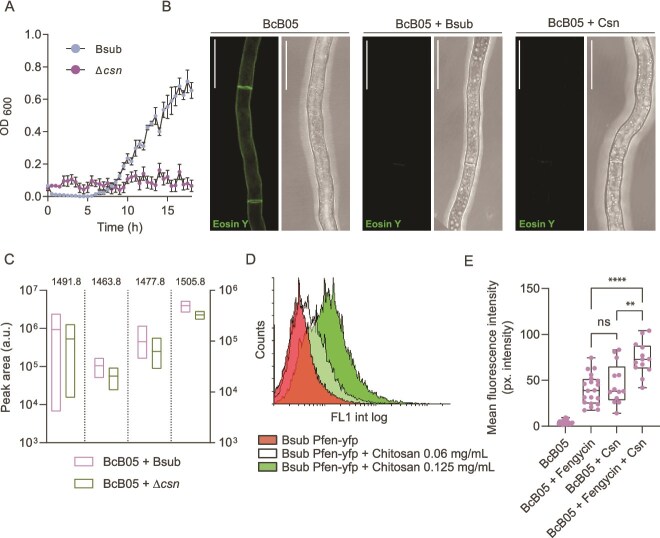
Chitosan degradation and the synergistic effects of chitosanase and fengycin contribute to *B. subtilis* growth. (A) Growth curve of the wild-type and ∆csn *B. subtilis* strains in minimal MSGG medium supplemented with chitosan (0.2 mg/ml) as the sole carbon source, showing no growth of the ∆csn mutant, highlighting the role of Csn in enabling *B. subtilis* to utilize chitosan. (B) Confocal microscopy images of *B. cinerea* hyphae stained with eosin Y to visualize chitosan. The fluorescence signal was absent in hyphae treated with wild-type *B. subtilis* or 0.2 mg/ml purified Csn, demonstrating chitosan degradation. The scale bar equals 20 μm. (C) Floating bar plot showing the peak abundances of four fengycin structural variants in the supernatant of *B. cinerea* after 24 h of interaction with wild-type *B. subtilis* or interaction with the ∆csn mutant. (D) Flow cytometry analysis of *B. subtilis* expressing YFP under the fengycin promoter (Bsub Pfen-yfp) in the presence of control conditions, 0.06 mg/ml chitosan, or 0.125 mg/ml chitosan, demonstrating chitosan-induced maintenance of the fengycin promoter over time. (E) Quantification of ROS intensity in *B. cinerea* treated with 10 μm fengycin, 0.2 mg/ml purified chitosanase, or a combination of both, demonstrating a synergistic effect between chitosanase and fengycin in inducing ROS accumulation. The whisker plot shows all the measurements (pink dots), medians (black line), and minimum and maximum values (whisker ends). In all experiments, at least three biological replicates are shown. Statistical significance was assessed via a t test, with double and quadruple asterisks indicating significant differences at *P* < .01 and *P* < .0001.

To test whether this highly unstable metabolite [84, 85] affects fungal growth, *Botrytis* cultures were treated with purified bacillaene at a concentration of 60 μg/ml. Hyphal tip splitting and massive cytoplasmic vacuolization were the most notable fungal morphological changes (Fig. 5B, Fig S12B). Furthermore, the external addition of purified bacillaene halted *Botrytis* growth, whereas the untreated *Botrytis* continued to grow, confirming the fungistatic role of bacillaene (Fig. 5C). Bacillaene is known to function as a bacteriostatic compound that inhibits bacterial protein synthesis, a mode of action consistent with its growth-inhibitory effects observed here on *B. cinerea* [86–88]. To investigate this further, *B. cinerea* cultures were treated with bacillaene for both short (24 h) and long (1 week) periods. Whole-genome sequencing of the long-term treated cultures revealed multiple mutations, including changes in the promoter region of a gene annotated as a cytoplasmic elongation factor (Bchbs1). However, additional mutations were also identified in genes associated with cell wall integrity, membrane remodeling, and oxidative stress responses (Table S4). These results suggest that *B. cinerea* may deploy a multifaceted adaptive response to counteract bacillaene toxicity, involving both transcriptional regulation and broader cellular stress pathways, rather than relying solely on modifications of the elongation machinery.

Bacillaene is a highly unstable molecule that is particularly sensitive to light and oxygen [84, 85], which raises the question of the mechanism that preserves the functionality of this ecologically significant molecule. *Bacillus* ECM is known to retain secondary metabolites [89], and we have shown that ECM components are upregulated during the interaction with *Botrytis*, two observations that led us to propose an ECM with a bacillaene-stabilizing role. TasA is capable of disorganizing the b-glycan layer and reaching the chitin-chitosan layer of the fungal cell wall, which further supports the hypothesis that TasA is a molecular vehicle for the stabilization and efficient delivery of bacillaene to the *Botrytis* hyphal surface. Molecular docking studies using the resolved crystal structure of TasA and bacillaene predicted the highly likely interaction of bacillaene with TasA (Fig. 5D). We performed native metabolomics experiments [90] to explore the interaction between TasA and the crude extract containing metabolites from *Bacillus–Botrytis* cocultures. Native metabolomics, performed by combining non-targeted metabolomics and native mass spectrometry, showed that the fold and function of TasA were preserved^73^. The crude metabolite extract was first separated via microflow UHPLC, and subsequently (post column), a make-up buffer neutralized the mobile phase, and purified TasA was infused via a T-splitter before MS analysis (Fig. 5E, left). Upon bacillaene passage through the system, the interaction with TasA was monitored via MS, which revealed a molecular mass shift corresponding to the formation of the TasA-bacillaene complex (Fig. 5E, right). This molecular mass shift, along with a distinct retention time, indicated the binding of bacillaene A, a version of bacillaene functionalized with a sugar residue in its structure, to TasA. To further support the proposed stabilizing role of TasA, we compared bacillaene levels in the supernatant of wild-type *B. subtilis* and a ΔtasA mutant after 24 h of culture. In the absence of TasA, bacillaene levels were drastically reduced, consistent with a protective or stabilizing role (Fig. S12C). Moreover, transcriptomic data from a previous RNA-seq study [49] showed that the ΔtasA mutant displays upregulation of the bacillaene biosynthetic operon (*pks*), likely as a compensatory response to reduced metabolite stability or extracellular accumulation. Although direct biophysical assays to quantify stabilization remain technically challenging due to the light and oxygen sensitivity of bacillaene, our native metabolomics and comparative expression analyses together support a model in which TasA contributes to preserving bacillaene bioavailability within the extracellular environment [49]. Therefore, we hypothesize that binding to TasA shields bacillaene A from degradation, enhancing its extracellular stability and facilitating its delivery to the fungal target. These findings suggest an unexpected functional link between biofilm matrix components and secondary metabolism, highlighting TasA not only as a structural ECM protein but also as a potential carrier and stabilizer of labile bioactive molecules during microbial interactions.

### *Bacillus subtilis* feeds on *Botrytis cinerea* chitosan and maintains fengycin production

The switch from fungicidal activity mediated by fengycin to fungistatic activity driven by bacillaene suggests a shift in the strategy adopted by *Bacillus* during its interaction with *Botrytis*. This change likely leads to long-term nutritional benefits, probably in oligotrophic environments, where maintaining a balance between microbial competition and coexistence could be advantageous for *B. subtilis*. In this context, the *B. subtilis* population density increased in coculture, mostly as vegetative cells and not spores, compared with monoculture (Fig. 1C). One explanation for these population dynamics is associated with the ability of *B. subtilis* cells to feed on *B. cinerea* hyphal structural components as nutrients. We hypothesize that chitosan, a key fungal cell wall polymer, serves as a carbon source for *B. subtilis* during interaction with *Botrytis*, supported by two complementary lines of evidence: first, the accessibility of *B. subtilis* cells to chitosan is mediated by the chemical affinity of TasA (Fig. 3D, Fig. S7A), and second, *B. subtilis* encodes a chitosanase (*csn*), an enzyme that hydrolyses the β-1,4-glycosidic bonds of chitosan molecules [91–93], enabling its utilization as a nutrient. The dynamic growth of bacteria in minimal MSGG medium with chitosan as the sole carbon source revealed the ability of the *B. subtilis* WT strain to grow; however, a ∆csn mutant strain lacking functional chitosanase was not able to grow (Fig. 6A). Further in situ degradation of fungal chitosan by *Bacillus* during the interaction with *Botrytis* was confirmed via the use of the chitosan dye eosin Y [94] and fluorescence microscopy (Fig. 6B). Furthermore, the addition of purified chitosanase to *B. cinerea* culture produced a similar reduction in the fluorescence signal of fungal hyphae, indicative of chitosan loss, underscoring its role in fungal cell wall breakdown (Fig. 6B). Metabolomic analysis further highlighted the role of chitosanase in the interaction between *B. subtilis* and *B. cinerea*. After 24 h of coculture, we observed a trend in the accumulation of fengycin isoforms, with lower amounts in the supernatant of cocultures with the ∆*csn* mutant than in that of cocultures with wild-type *B. subtilis* (Fig. 6C). This result suggests a link between chitosanase activity, or the degradation of chitosan, and fengycin production. Flow cytometry analysis further supported this connection, revealing heightened fengycin promoter activity in the presence of chitosan after 1 week, which suggests that *B. cinerea* cell wall components sustain expression over time (Fig. 6D) and explains why, in the interaction, without accessible chitosan, fengycin accumulation is reduced. This result also suggested the possibility of a synergistic effect between Csn and fengycin against *Botrytis*. Compared with single treatments, the application of purified Csn and fengycin at the same time to *B. cinerea* significantly increased ROS production (Fig. 6E). These findings suggest that *B. subtilis* employs a dual strategy, nutrient acquisition and metabolic modulation, through both physical and chemical means to keep *B. cinerea* in a slowed growth state, ensuring the sustained availability of a carbon source in long-term balanced antagonistic coexistence.

## Discussion

**Figure 7:**
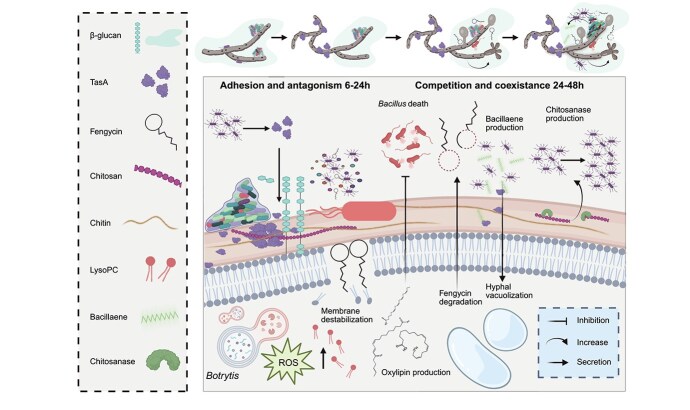
Overview of the dynamic interaction and counter defense between *B. subtilis* and *B. cinerea*. The interaction between *Bacillus* and *Botrytis* is a highly dynamic process characterized by mutual adaptation. *Bacillus* ECM components, specifically TasA and fengycin, induce stress in *B. cinerea*, which responds by forming chlamydospores, secreting oxylipins and neutralizing fengycin. In turn, *Bacillus* has shifted its strategy, producing bacillaene to target fungal mitochondria. Additionally, *Bacillus* utilizes chitosan from the fungal cell wall, resulting in ecological competition between the two organisms.

In this study, we demonstrated that the *B. subtilis* ECM, in addition to playing a structural role in the communication with other bacteria and plants [13, 14], is essential for fungal hyphal colonization and antagonism against pathogenic fungi (Fig. 7). Our observations indicate that the ECM can play an offensive or defensive role in interspecies interactions, depending on the competitor. In interactions with *Pseudomonas*, defense seems to be the prevalent function [14]; however, in interactions with *B. cinerea*, the role of ECM is more offensive. We show that the ECM component TasA and the matrix-associated factor fengycin are crucial for antagonizing *B. cinerea*, acting synergistically while playing complementary roles, with each modifying *B. cinerea* physiology differently and triggering distinct metabolic changes.

Our data revealed that the ECM, specifically TasA, not only facilitates physical attachment but also orchestrates metabolite-driven antagonistic mechanisms, positioning the *B. subtilis* ECM as an active mediator of microbial competition. We specifically propose that the ECM component TasA has two unprecedented functions: one in altering the physiology of *B. cinerea* hyphae and the other as a putative stabilizer and carrier of the bioactive and highly sensitive molecule bacillaene. TasA alone directly disrupts *Botrytis* physiology by disorganizing its β-glucan layer, resulting in cellular and morphological damage. Moreover, our results highlight the specificity of fungal defenses against *B. subtilis* lipopeptides. For example, surfactin has a lethal effect on *Aspergillus*, inducing chlamydospore formation; however, it does not trigger this response in *B. cinerea* [16]. Conversely, fengycin has no apparent effect on the lifestyle of *Aspergillus* but does induce chlamydospore formation in *B. cinerea*. These findings suggest that the effectiveness of *B. subtilis* lipopeptides may be highly dependent on the unique membrane composition of different fungal species.

Our results illustrate the complexity of the *Bacillus–Botrytis* interaction, which involves bidirectional responses and dynamic chemical interplay, challenging the notion of purely antagonistic interkingdom bacteria–fungi interactions. Although *B. subtilis* initially suppresses fungal growth, the *B. cinerea* population initiates a chemical defense response by producing oxylipins that target *B. subtilis* and restructuring its cell morphology through hyperbranching and chlamydospore formation to increase survival. Some oxylipins, such as SDA, have been previously identified as mediators of survival strategies produced by the eukaryote *Caenorhabditis* elegans coexisting with *Pseudomonas* [95], suggesting a conserved resilience mechanism among eukaryotes. The accumulation of Lyso-PC and ROS in *Botrytis* in response to TasA further indicates the complex biochemical interaction and metabolomic modulation of *B. cinerea* by *B. subtilis* ECM. Lyso-PC is implicated in oxidative stress in various organisms, and its accumulation in *B. cinerea*, alongside ROS, suggests the existence of a defense mechanism activated by the fungal cell in response to *B. subtilis* ECM. As part of this defense, *B. cinerea* partially neutralizes *B. subtilis* action by degrading fengycin, as previously reported for bacteria–bacteria interactions [82, 96] but not for interactions with fungi. After 24 h of interaction, the production of another secondary metabolite from the *Bacillus* arsenal, bacillaene, increases. The role of bacillaene, which functions fungistatically rather than as a fungicide, exemplifies this adaptive strategy. Known to mediate microbe–microbe interactions [97] and plant interactions*,* increased bacillaene production in response to fungal degradation of fengycin reflects the adaptability of *Bacillus* chemical weapons in response to *B. cinerea* counterattack. Bacillaene has been shown to inhibit bacterial protein synthesis by targeting the elongation factor FusA, and a similar mechanism in fungi cannot be ruled out. Sequence analysis revealed partial homology between FusA and two *B. cinerea* elongation factors (BcMef1 and Bchbs1), and *in silico* docking suggested a potential interaction of bacillaene with these proteins (Fig. S13A). After prolonged exposure to bacillaene, a mutation was detected in the promoter region of Bchbs1, a finding that may reflect transcriptional adaptation. Although these data point toward a possible link between bacillaene and the fungal translation machinery, this remains speculative and will require future biochemical and functional validation. Whole-genome sequencing additionally revealed mutations in genes unrelated to translation, including those associated with cell wall remodeling, redox homeostasis, and stress signaling pathways. This broader transcriptional and genomic remodeling suggests that *B. cinerea* does not respond through a single resistance mechanism, but rather engages a multifactorial adaptation strategy. Such a pattern is consistent with a fungistatic activity of bacillaene, in which the compound halts fungal growth and triggers cellular stress without causing rapid cell death. Therefore, although bacillaene exerts a clear inhibitory effect, its precise mode of action in fungi likely involves multiple physiological targets and warrants further investigation.

We demonstrated that TasA binds to and stabilizes bacillaene, likely contributing to the fungistatic functionality. A previous study demonstrated the ability of bacillaene to bind the E. coli curli protein components CsgA and CsgB [41], but in this case, it disrupted the assembly of amyloid fibers and thus biofilm formation, a strategy that confers *B. subtilis* with a competitive advantage in microbial interactions.

In the sequence of events leading to this interkingdom interaction, it may be predicted that following attachment to *B. cinerea* via TasA, *Bacillus* employs chitosanase to degrade chitosan from the *B. cinerea* cell wall and gain access to an extra carbon source. This ability to exploit *B. cinerea* for carbon is demonstrated by increased *B. subtilis* growth in coculture. Our results show that chitosanase acts synergistically with fengycin, increasing cell wall susceptibility to further fungal stress. Flow cytometry indicated that the presence of chitosan extends fengycin promoter activity, potentially stabilizing the antifungal arsenal of *B. subtilis*. Thus, the ECM enables *B. subtilis* to sustain a fungistatic hold over *B. cinerea*, maintaining competitive dominance while avoiding ecological disruption through complete eradication.

In short-term interactions, these adaptive responses may reflect a shift toward a stable antagonistic coexistence, consistent with ecological theories on microbial competition, suggesting that in some antagonistic interactions, competitors may reach a nonlethal equilibrium where metabolic adjustments allow for prolonged coculture [6, 47]. This adaptability may exemplify a broader fungal resilience strategy, wherein *B. cinerea* perceives specific ECM components from *B. subtilis*, thereby modulating its physiological defenses and enhancing survival. Competition upon initial contact can often evolve into a form of stable coexistence, where both *B. subtilis* and *B. cinerea* adapt and survive despite ongoing antagonism. Such coexistence may lead to spatial or resource partitioning, ultimately enabling both organisms to persist within a shared niche.

In conclusion, our data expand the understanding of microbial interactions by revealing the role of the bacterial ECM in mediating physical attachment and extracellular matrix-driven antagonism, with implications for microbial competition and survival strategies in resource-limited environments. Through such interactions, the *B. subtilis* ECM has emerged as a central element in microbial antagonism, shaping microbial interplay and sustaining *B. subtilis* survival under competitive conditions.

## Supplementary Material

Supplementary_materials_clean_wraf277

Table_S1_20251203_wraf277

Table_S2_20251203_wraf277

Table_S3_20251203_wraf277

Table_S4_20251203_wraf277

Supplementary_materials_wraf277

## Data Availability

All the raw RNA-seq data have been submitted to the Gene Expression Omnibus (GEO) and can be accessed through GEO series accession no. GSE287578 (URL: https://www.ncbi.nlm.nih.gov/geo/query/acc.cgi?acc=GSE287578. All the metabolomics data are deposited at https://massive.ucsd.edu/with the identifier MSV000089552.
