# Supplementary material for: Offensive role of the Bacillus extracellular matrix in driving metabolite-mediated dialog and adaptive strategies with the fungus Botrytis
Source: ISME J. 2025 Dec 18;19(1):wraf277. doi: 10.1093/ismejo/wraf277 (PMC12771376; doi:10.1093/ismejo/wraf277)
Supplement: Supplementary_materials_wraf277 [file supplementary_materials_wraf277.pdf]

Supplementary material

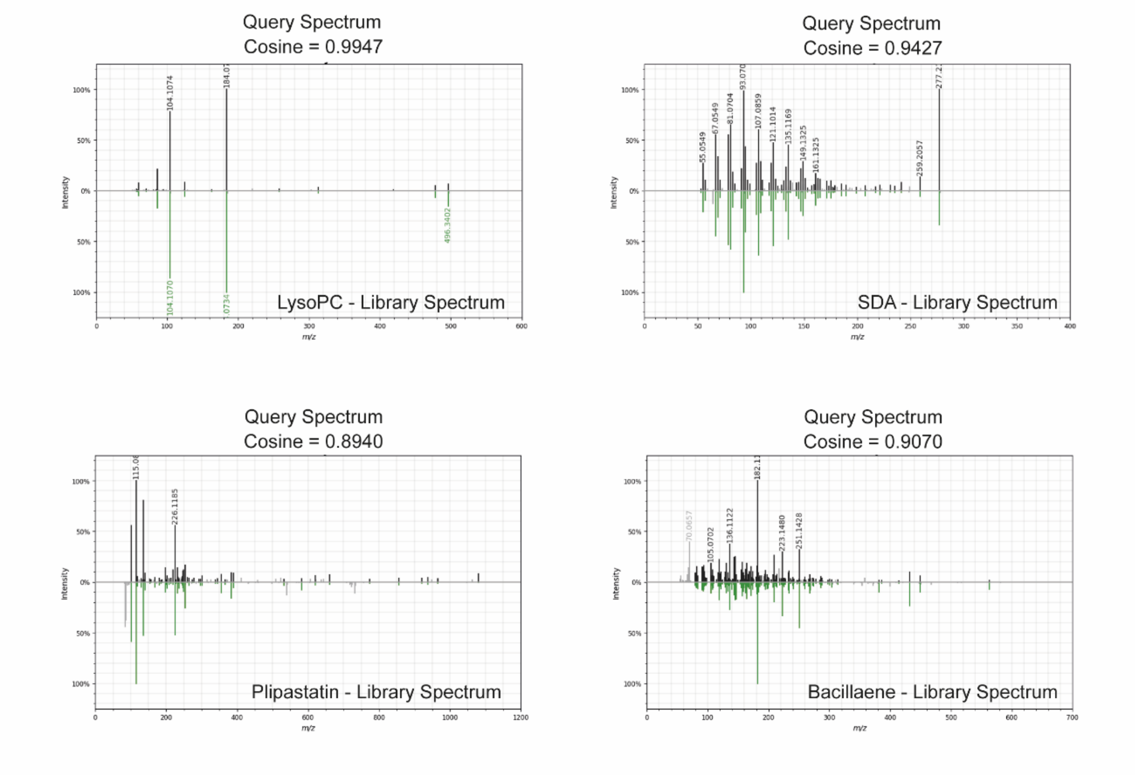

Figure S1. Mirror plots comparing spectra from named features throughout the manuscript to standard spectra deposited in GNPS. The upper part of the plot (black lines) represents the MS spectra of the candidate feature, and the lower part (green lines) represents the MS spectra of the standard compound. Mirror plots were generated using https://metabolomics-usi.ucsd.edu/.

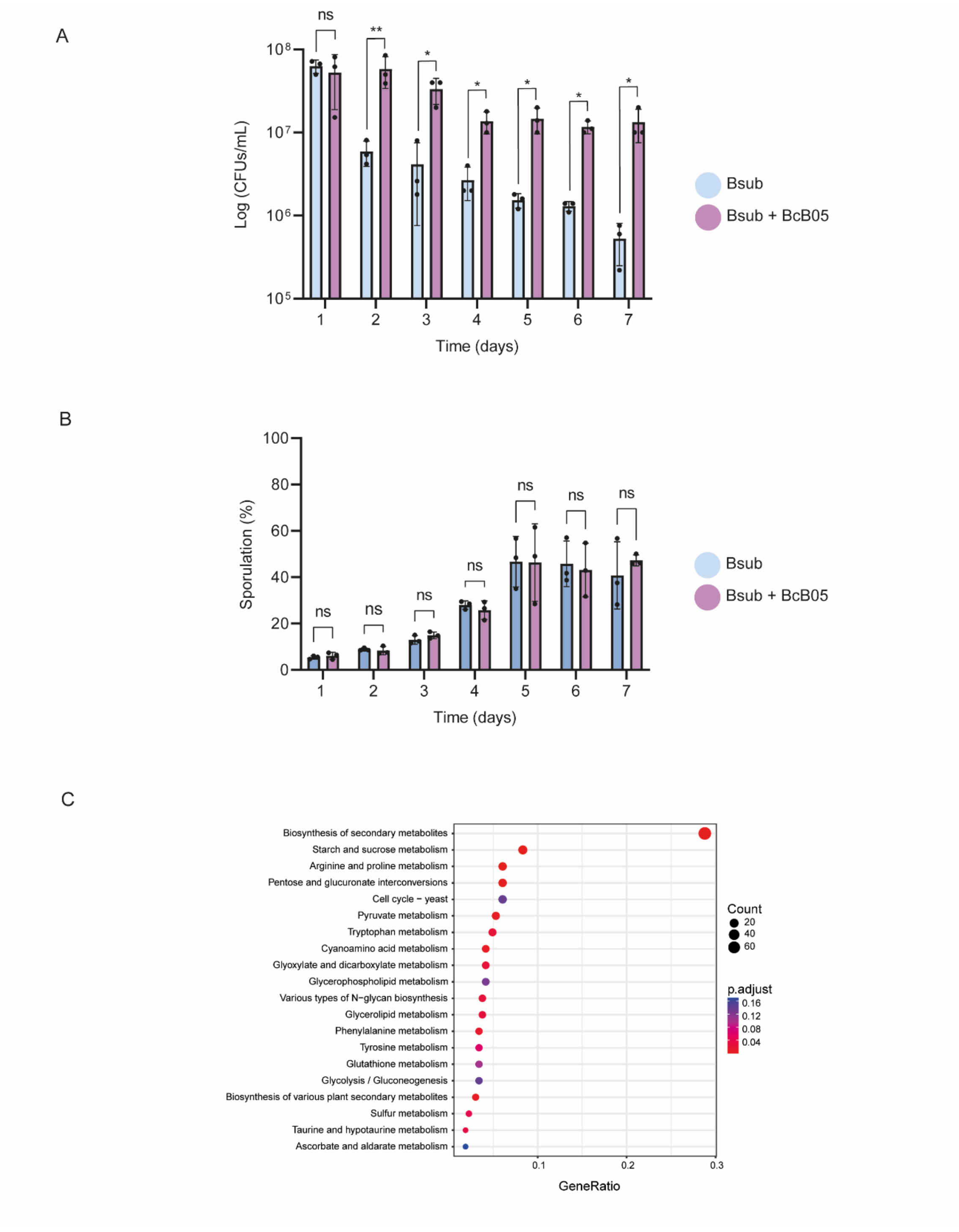

Figure S2. Growth dynamics of *Bacillus subtilis* and transcriptional response of *Botrytis cinerea* during coculture. A) Time-course of *Bacillus* CFUs over 7 days in monoculture (blue) and coculture with *B. cinerea* (pink). **B)** Percentage of *Bacillus* spores relative to total viable cells in monoculture (blue) and coculture with *B. cinerea* (pink). Statistical significance was assessed via a *t test*, with double asterisks indicating significant differences at P < 0.01. **C) Enrichment analysis of differentially expressed *B. cinerea* genes during**

interaction with *B. subtilis.* KEGG pathway enrichment analysis showing the deregulation of pathways related to glutathione metabolism, secondary metabolite biosynthesis, and phospholipid metabolism after six hours of coculture.

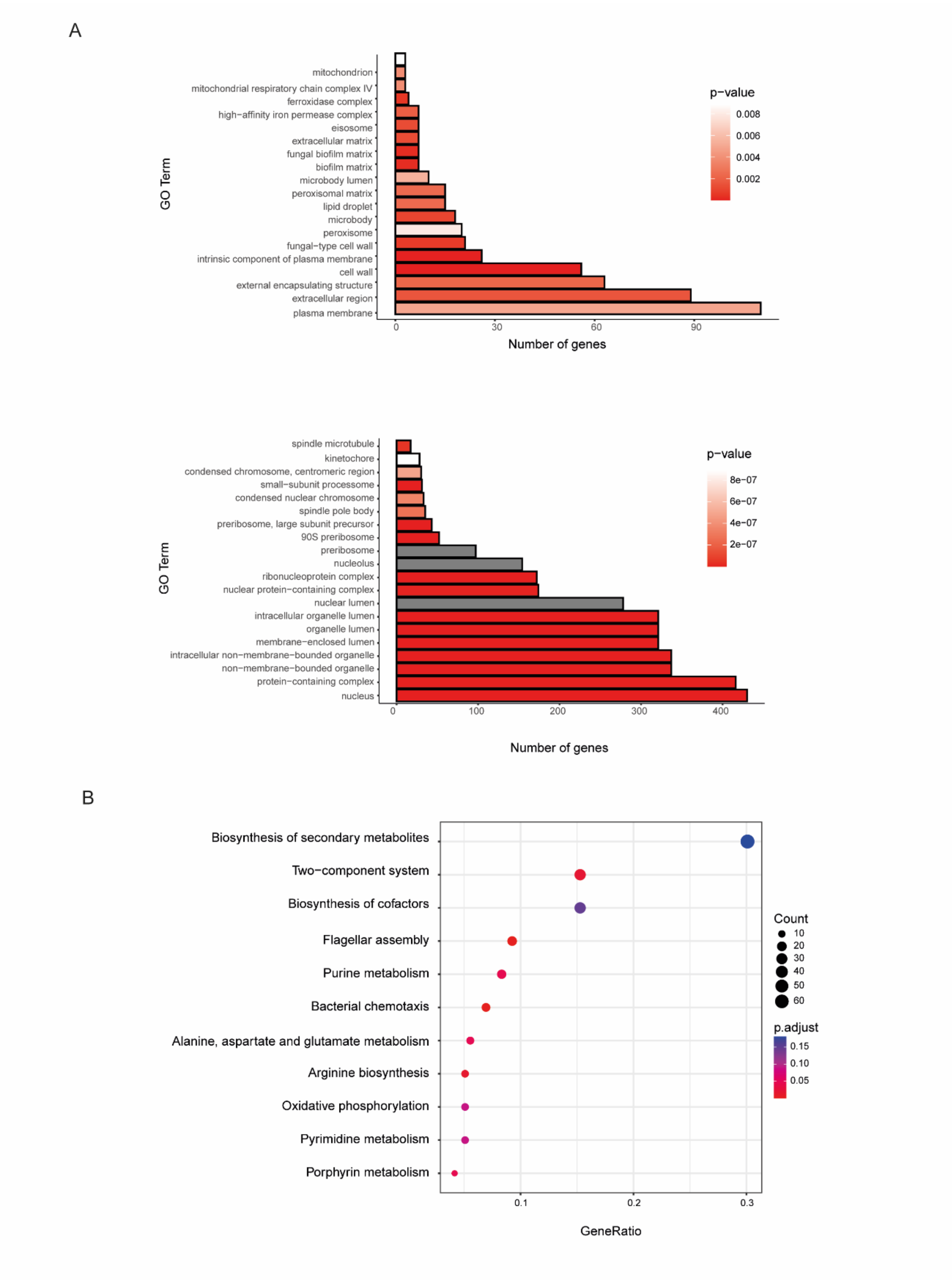

Figure S3. Functional enrichment analyses of *B. cinerea* and *B. subtilis* genes during coculture. A) Gene Ontology (GO) terms enriched in genes upregulated in *B. cinerea* after six hours of coculture. **B)** GO terms enriched by genes downregulated in *B. cinerea* after six hours of coculture. **C) Enrichment analysis of differentially expressed *B. subtilis* genes during interaction with**

B. cinerea**.** KEGG pathway enrichment analysis showing the deregulation of pathways related to the biosynthesis of secondary metabolites.

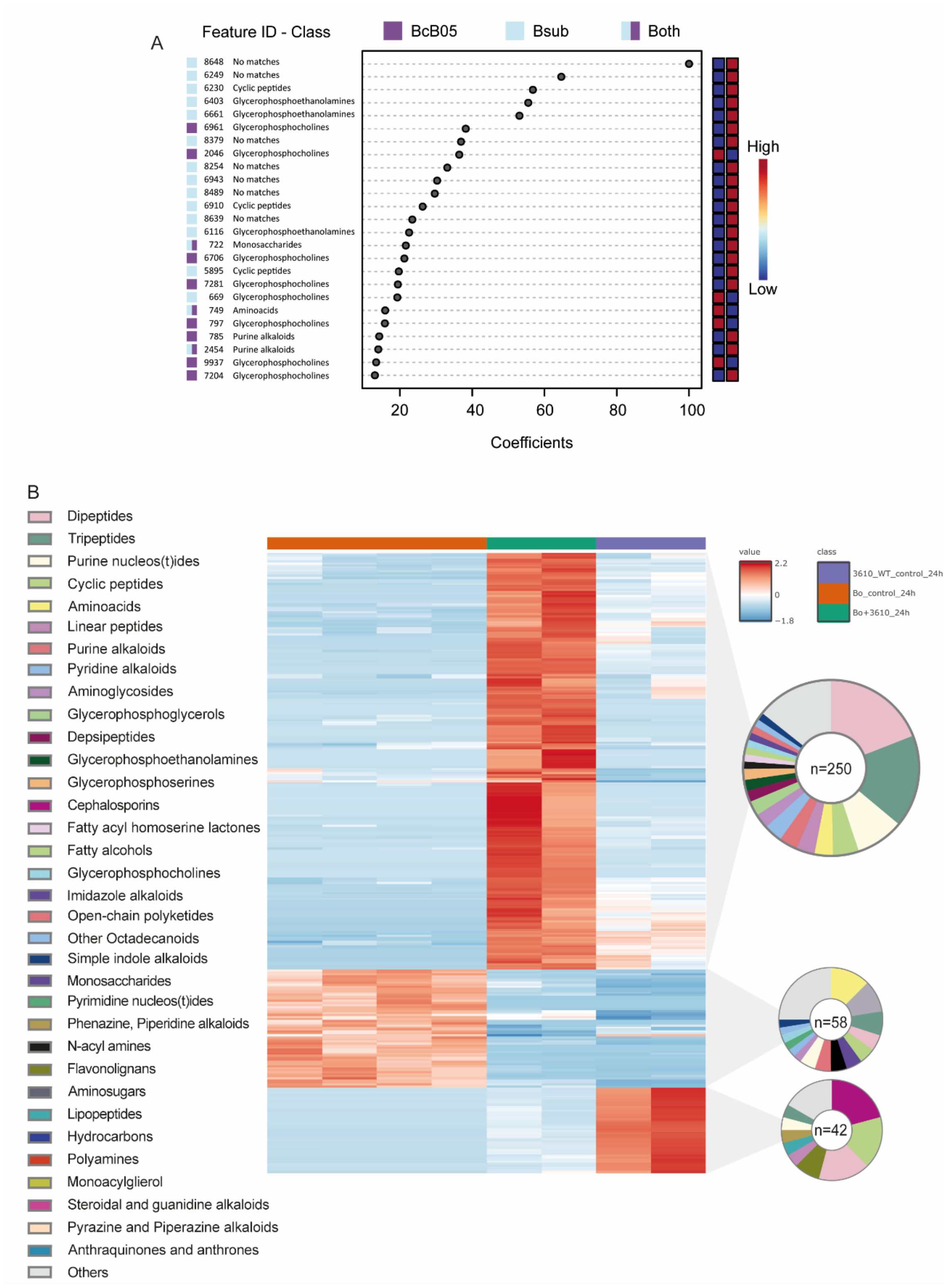

Figure S4. Metabolomic analysis revealed differential accumulation of metabolite classes during the *Bacillus-Botrytis* interaction. A) Top 25 features with the highest median weighted sum of absolute regression coefficient scores determined by PLS-DA, calculated using MetaboAnalyst. These features were identified as key metabolites discriminating cocultures from single cultures

of each microbe after 24 h in the cell fraction. The feature IDs are annotated with their chemical classes via Classyfire classification. The left side of the figure indicates the microbial origin of the different accumulated metabolite classes. **B)** Heatmap of hierarchical clustering of the top 350 features within impacted molecular families during the coculture of *B. cinerea* and *B. subtilis* compared with monoculture of each after 24 h in the cell fraction. The color gradient in the heatmap indicates the relative fold change of each metabolite between the groups. The circular graphs show the percentage distribution of metabolite classes with altered accumulation, with the color code inside the donut chart representing each chemical class according to NPClassifier.

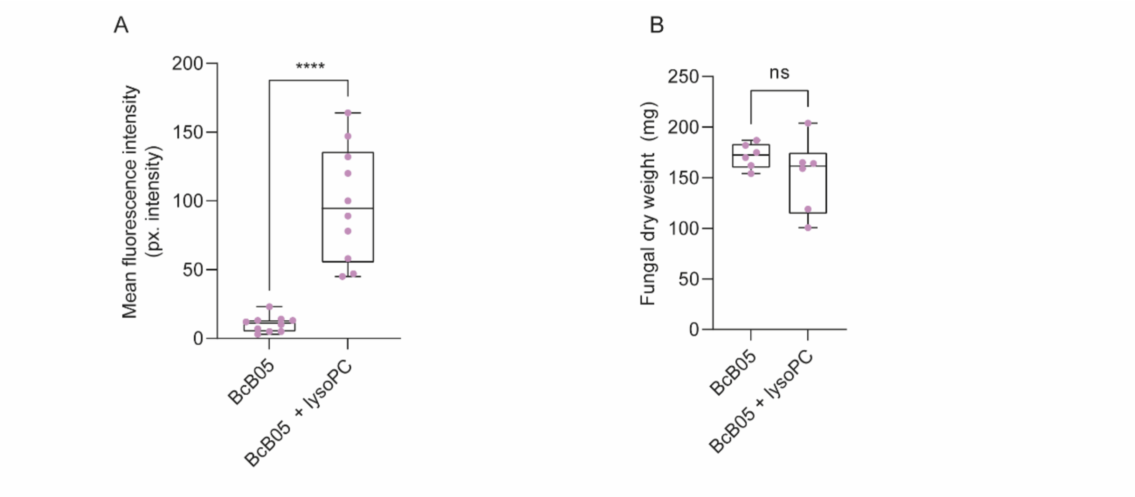

Figure S5. Lyso-PC induces ROS without affecting *B. cinerea* growth. A) Quantification of ROS levels (stained with DHR123) in *B. cinerea* after treatment with 150 µM lysoPC or no treatment for 24 h. The whisker plot shows all the measurements (pink dots), medians (black line), and minimum and maximum values (whisker ends). For all experiments, the results from at least three biological replicates are shown. Statistical significance was assessed via a t test, with quadruple asterisks indicating significant differences at $P < 0.0001$. **B)** Fungal growth of *B. cinerea* treated with 150 µM commercial lyso-PC and control *Botrytis*, showing that lyso-PC-induced ROS do not lead to *B. cinerea* growth inhibition. The whisker plot shows all the measurements (pink dots), medians (black line), and minimum and maximum values (whisker ends). For all experiments, the results of at least three biological replicates are shown. Statistical significance was assessed via t test.

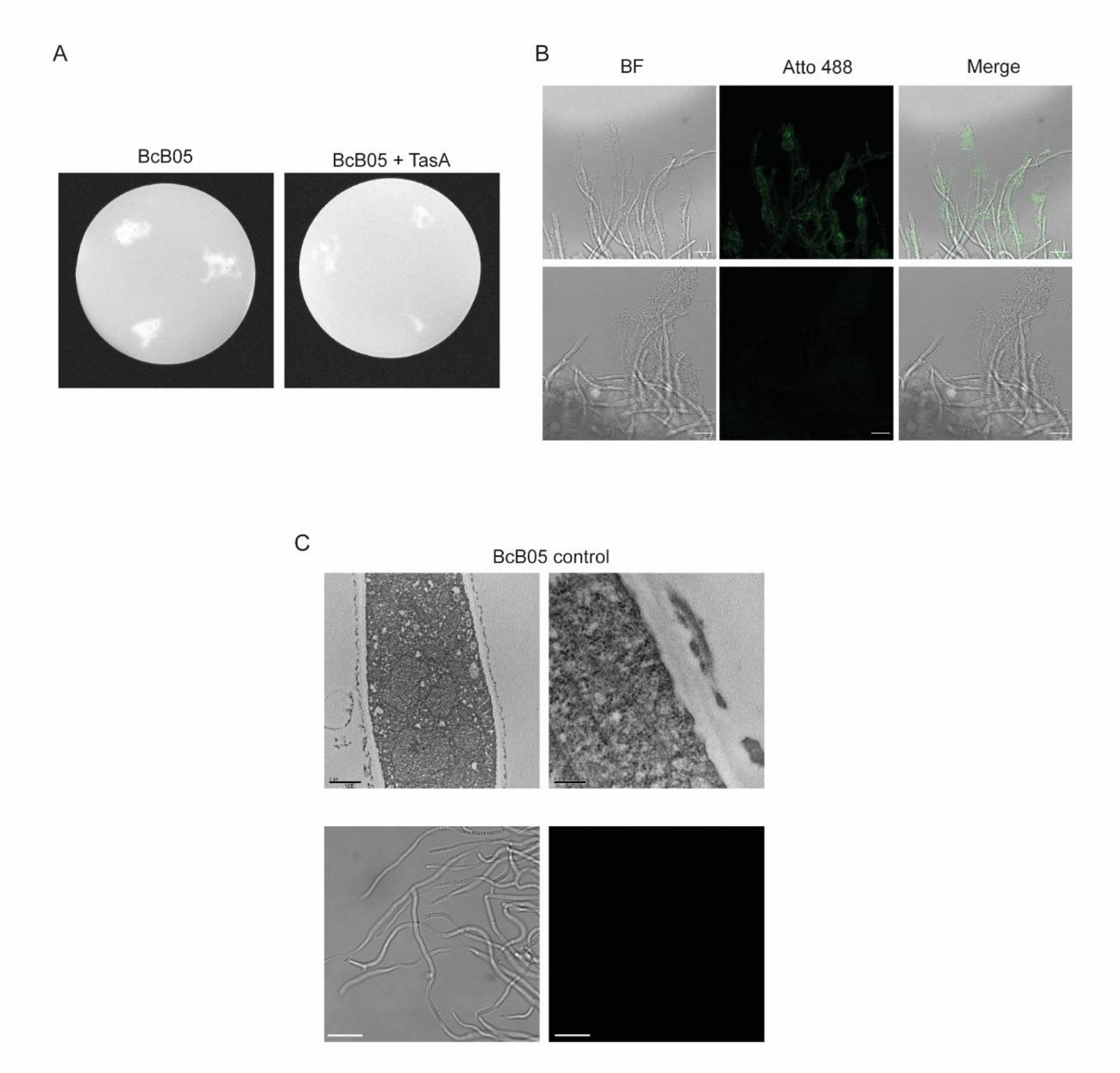

Figure S6. Imaging analyses of *B. cinerea* macrocolonies and TasA localization. A) Bright field images of *B. cinerea* macrocolonies corresponding to the MRI analysis. Each image shows three fungal microcolonies **B)** Immunofluorescence microscopy showing TasA localization on *Botrytis* hyphae during co-culture with *B. subtilis*. The top row shows samples stained with anti-TasA primary antibody followed by fluorescent secondary antibody, revealing TasA-associated signal on the fungal surface. The bottom row shows the negative control, where only the secondary antibody was applied (no primary antobody), confirming the specificity of the signal. **C) Top:** Transmission electron micrograph of negatively stained thin sections of untreated *B. cinerea* hyphae with immunogold labelling. The scale bar represents 1 μm for the image on the left and 0.2 μm for the zoomed-in image on the right. **Bottom:** Immunocytochemistry of *Botrytis* hyphae treated with TasA without anti-TasA

antibodies but with secondary antibodies as a control. Scale bars represent 20 μm.

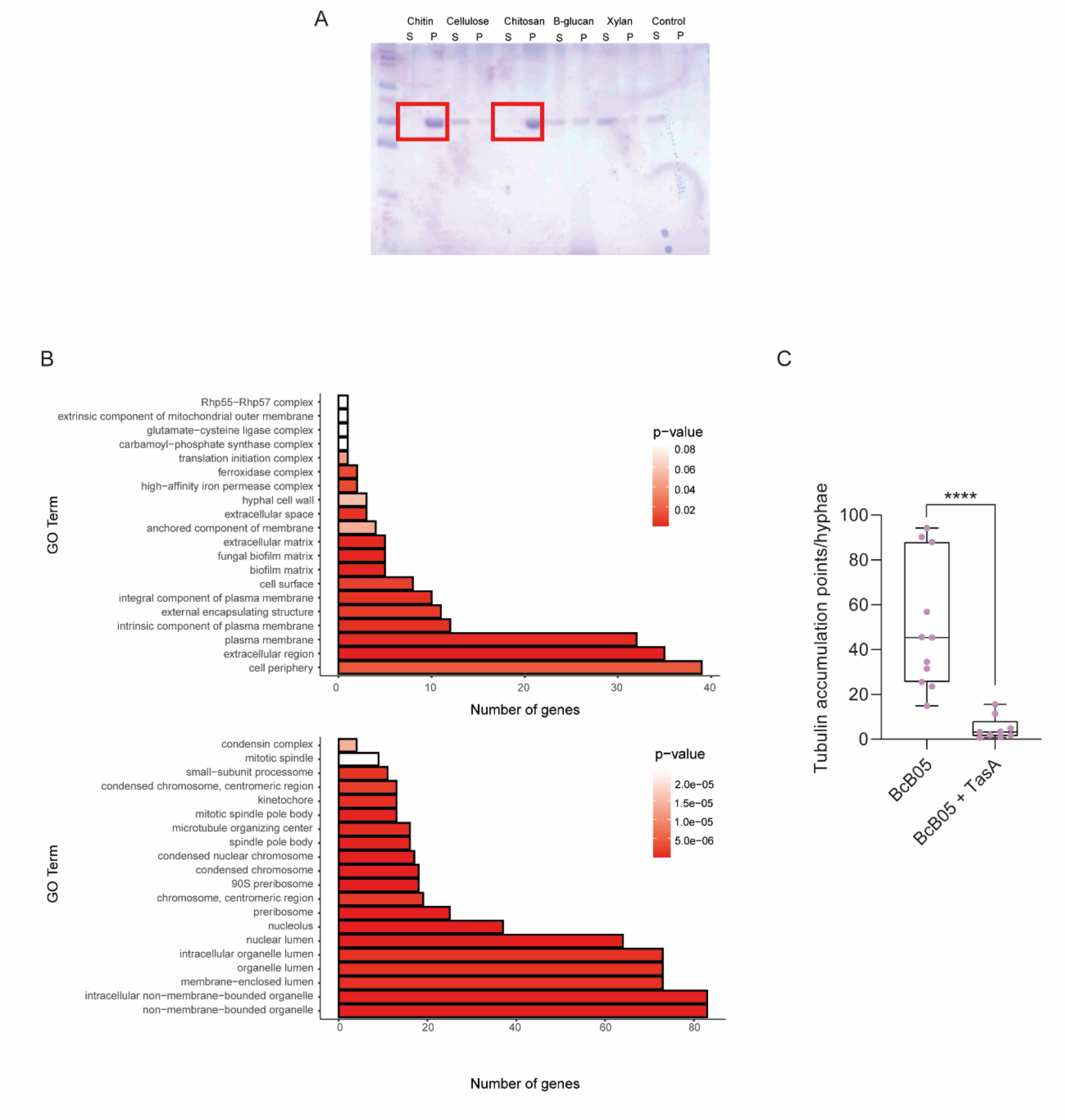

Figure S7. Cellular responses of *B. cinerea* to TasA treatment. A) Coomassie gel of polysaccharide affinity assays with purified TasA. **B)** Enrichment analysis of differentially expressed *B. cinerea* genes after six hours of treatment with 3 µm purified TasA. GO terms enriched by upregulated (top) and downregulated (bottom) genes of *B. cinerea* after treatment with TasA. **C**) Quantification of tubulin accumulation in pear hyphae treated with 3 µm purified TasA or left untreated. The whisker plot shows all the measurements (pink dots), medians (black line), and minimum and maximum values (whisker ends). For all experiments, the results from at least three biological replicates are shown. Statistical significance was assessed via a t test, with quadruple asterisks indicating significant differences at $P < 0.0001$.

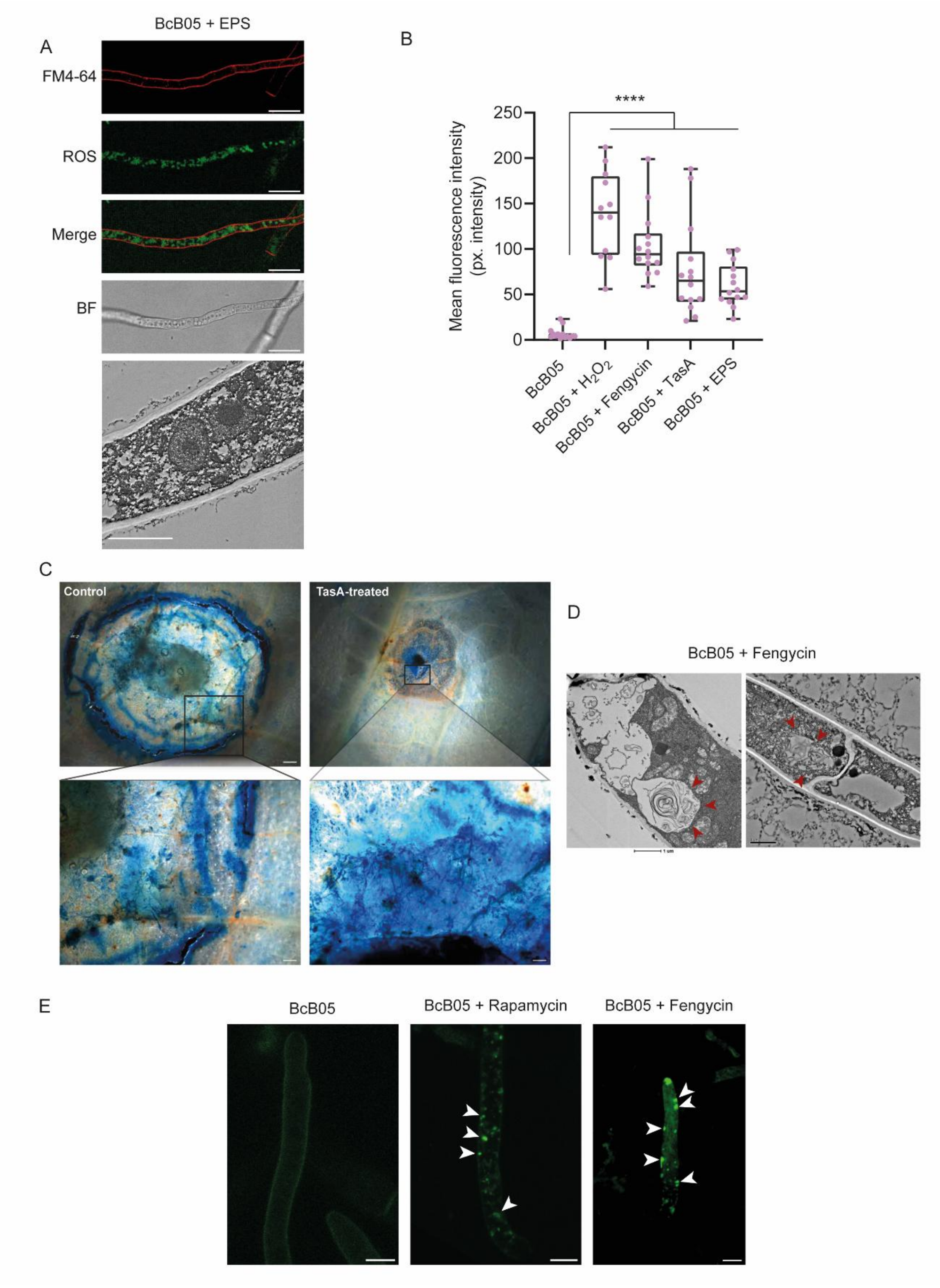

Figure S8. Differential effects of EPS and fengycin on *B. cinerea* physiology and stress responses. A) Representative confocal microscopy images showing ROS accumulation and the fungal membrane (stained with DHR123 and FM4-

64, respectively) in *B. cinerea* hyphae treated with EPS. Scale bars represent 20 µm. The lower panels show transmission micrographs of *B. cinerea* treated with 0.5 mg/mL purified EPS, revealing nonpronounced ultrastructural disorganization or autophagosome formation. Scale bars represent 2 µm. **B)** Quantification of ROS levels (stained with DHR123) in *B. cinerea* without treatment, with H_2O_2 as positive control, after treatment with 3 µM TasA, 0.5 mg/mL EPS or 10 µM fengycin for 24 h. Whisker plot shows all measurements (pink dots), medians (black line), and minimum and maximum (whiskers ends). In all experiments, at least three biological replicates are shown. Statistical significance was assessed ANOVA by one-way ANOVA with Dunnett's multiple comparisons test (each treatment vs BcB05 control), with quadruple asterisk indicating significant differences at $P < 0.0001$. **C)** Optical microscopy of *B. cinerea*-induced lesions on melon leaves. Infected tissues were stained with Cotton Blue to visualize fungal structures. Control infections showed extensive necrosis with thin, regularly branched hyphae penetrating mesophyll tissue. TasA-treated infections exhibited smaller lesions and abnormal hyphae with thickened walls, apical swelling, and restricted penetration, consistent with direct TasA-induced antifungal damage. **D**) Representative TEM images of *B. cinerea* treated with 10 µM fengycin for 24 hours. Autophagosomes are indicated with red head arrows. Scale bars are included in the images. **E)** Formation of autophagosomes in *Botrytis cinerea* after treatment with rapamycin or fengycin. Fluorescent punctate structures corresponding to autophagosomes were visualized using DAPGreen staining. An increased number and intensity of fluorescent spots were observed in treated samples, indicating the induction of autophagy. Scale bars equal 5 µm.

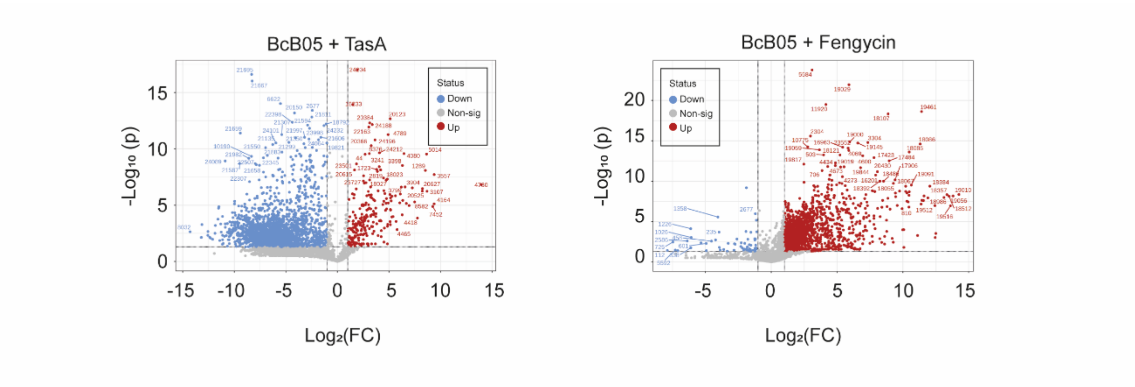

Figure S9. Differentially abundant metabolite accumulation in *B. cinerea* supernatants after TasA and fengycin treatments. Volcano plots showing metabolites with significantly greater or lower accumulation in the *B. cinerea* supernatant after 24 h of treatment with TasA (left) or fengycin (right) than in the supernatant of untreated *B. cinerea*. Analyses were performed using MetaboAnalyst.

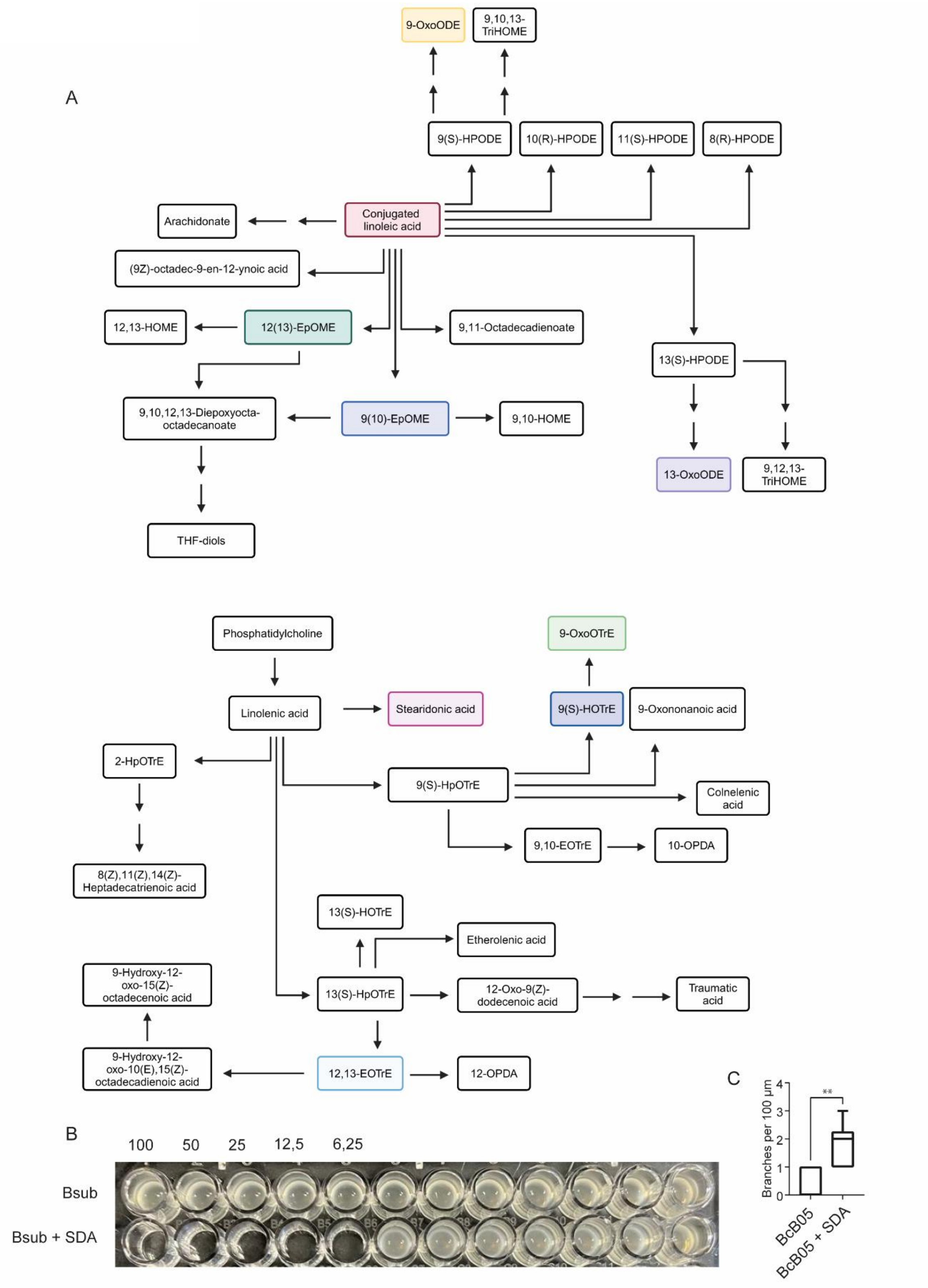

Figure S10. KEGG pathway-based schematics of oxylipins accumulated in *Botrytis cinerea* supernatant after treatment with fengycin. A) Two pathway diagrams representing the accumulation of oxylipins derived from linoleic (top scheme) and linolenic (bottom scheme) acids in the *B. cinerea* supernatant. The

accumulated metabolites are colored according to the same scheme as the border node color in **Figure 4A**. **B)** MIC assay of *B.subtilis* growth in response to SDA. Top row: *B. subtilis* growth as control in LB medium with DMSO. Bottom row: growth of *B. subtilis* with decreasing concentration of SDA (µg/mL). **C)** Quantification of hyphal branching in *B. cinerea* cultures treated with 6,25 µg/mL SDA compared to untreated controls. Data are expressed as the number of lateral and apical branches per 100 µm of hyphal length. Measurements were performed on six independent samples, with different microscopic fields analyzed per sample.

m/z 755.9276; M=1508

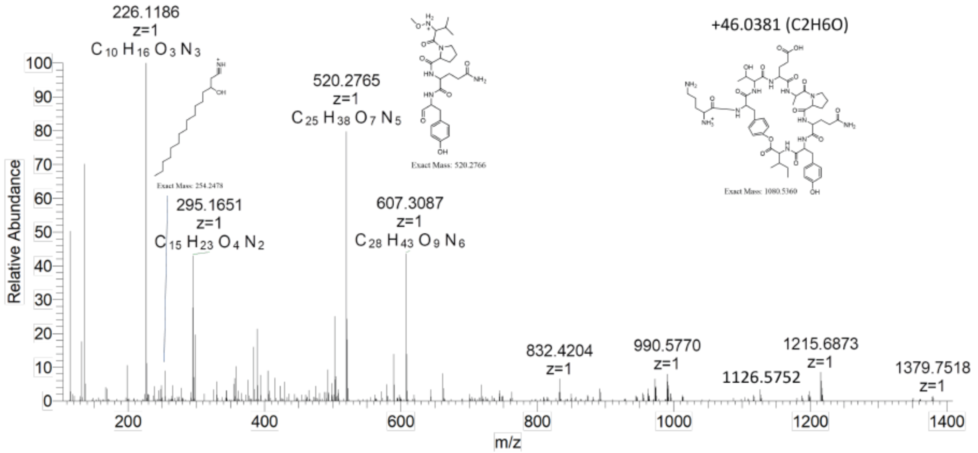

m/z 748.4184; M=1494

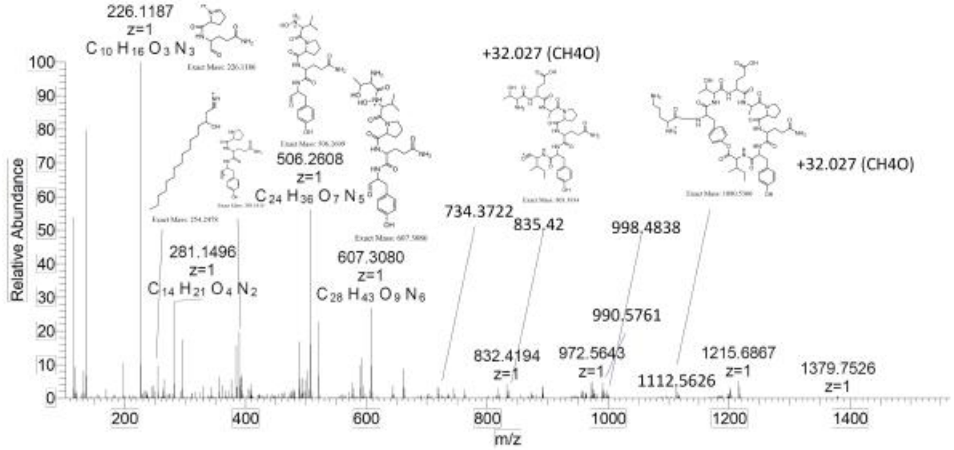

m/z 755.9276; M=1508

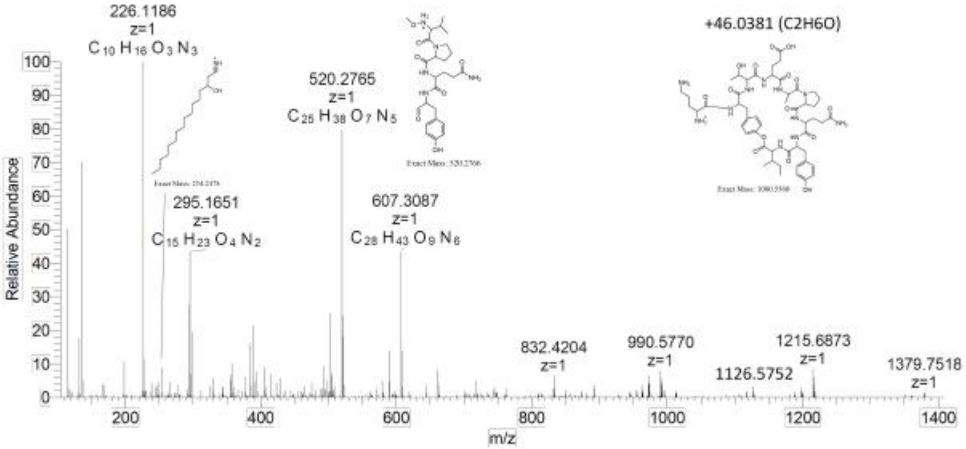

Figure S11. Targeted LC–MS/MS analysis of fengycin variants detected during interaction with *B. cinerea*. Upper panel: Reference MS/MS spectrum of fengycin C_{16}A (m/z 732.4053, z = 2). **Middle panel**: MS/MS spectrum of the 1494 Da variant (m/z = 748.4184, z = 2). **Bottom panel:** MS/MS spectrum of the 1508 Da variant (m/z = 755.9576, z = 2).

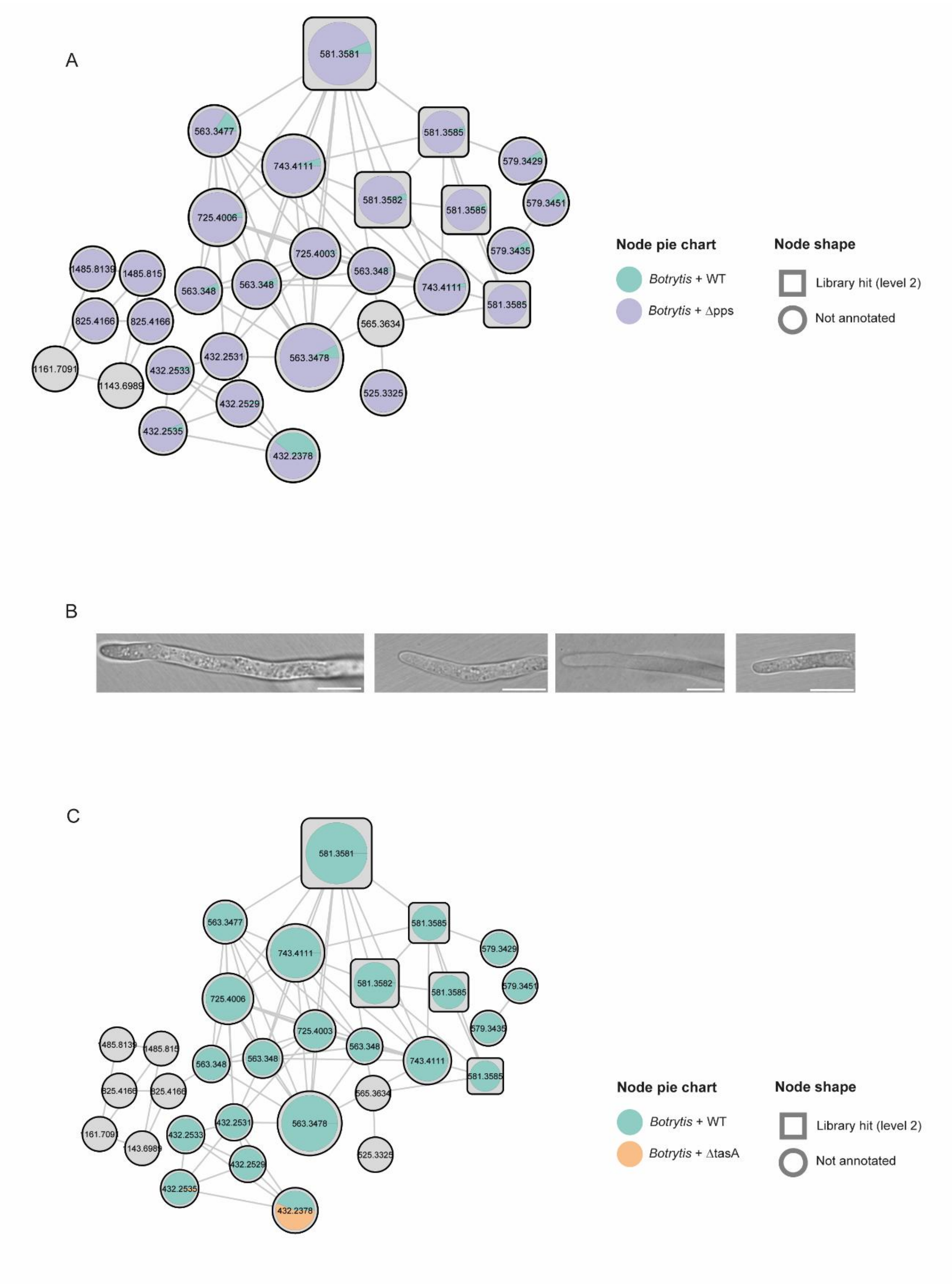

Figure S12. A) Molecular family analysis of bacillaene in *B. cinerea* supernatant after interaction with wild-type *B. subtilis* compared with *B. cinerea* interacting with ∆pps after 48h. Pie charts represent the mean peak abundance of metabolites in the supernatant, with node shapes indicating the level of metabolite identification according to GNPS libraries. **B)** Representative

confocal microscopy images of the tips of untreated *B. cinerea* hyphae. Scale bars equal 10 µm. **C)** Molecular family analysis of bacillaene in *B. cinerea* supernatant after interaction with wild-type *B. subtilis* compared with *B. cinerea* interacting with ΔtasA after 24h. Pie charts represent the mean peak abundance of metabolites in the supernatant, with node shapes indicating the level of metabolite identification according to GNPS libraries.

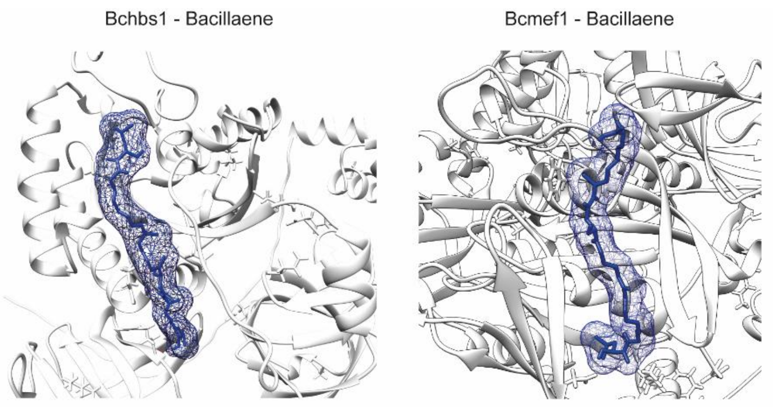

Figure S13. Molecular docking analysis predicted a putative binding site between bacillaene and the cytoplasmic elongation factor Bchbs1, as well as between bacillaene and the mitochondrial elongation factor Bcmef1, in *Botrytis cinerea*.

Table S1. List of strains used in this study.

Table S2. Named features throughout the manuscript and their parameters for identification within the framework of Sumner *et al.*, 2007.

Table S3. *B. cinerea* B05.10 putative secreted proteins upregulated upon challenging with Fengycin.

Table S4. Full List of mutated genes in *Botrytis cinerea* treated with bacillaene for one week compared with the *Botrytis* control. Across the set of *Botrytis* genes carrying variants after long-term co-incubation with *Bacillus subtilis,* we observe a coherent functional signature centered on (i) cell-envelope remodeling and glycan modification (CAP10-like, CBM21, Sun/CE16, WSC, CFEM), (ii) reprogramming of solute/metal transport including MFS, AQP2, and a ferric-chelate reductase and (iii) activation of signaling and genome-maintenance axes (Tel1/ATM-like, ABC1-like, calcineurin-like). Additional changes implicate broad transcriptional control (Zn(2)-C6, AreA), proteostasis via ubiquitination (RING/F-box), and post-transcriptional regulation (Argonaute/NTC-related). Notably, most variants localize to UTRs and promoter-proximal regions, indicating adaptation primarily through expression modulation rather than widespread structural protein alteration; the few high-impact coding changes (e.g., frameshift in BCIN_10g06270 and stop-gained in the NACHT protein) are consistent with tuning of surveillance/stress pathways. Overall, the data support an adaptive program expected under nutrient competition and antimicrobial pressure, whereby *Botrytis* strengthens its barrier and transport systems, optimizes resource uptake, and buffers oxidative/osmotic stress.
